# Structural basis of broad-spectrum β-lactam resistance in *Staphylococcus aureus*

**DOI:** 10.1038/s41586-022-05583-3

**Published:** 2023-01-04

**Authors:** J. Andrew N. Alexander, Liam J. Worrall, Jinhong Hu, Marija Vuckovic, Nidhi Satishkumar, Raymond Poon, Solmaz Sobhanifar, Federico I. Rosell, Joshua Jenkins, Daniel Chiang, Wesley A. Mosimann, Henry F. Chambers, Mark Paetzel, Som S. Chatterjee, Natalie C. J. Strynadka

**Affiliations:** 1 Department of Biochemistry and Molecular Biology, The University of British Columbia, Vancouver, British Columbia Canada; 2 Centre for Blood Research, The University of British Columbia, Vancouver, British Columbia Canada; 3 HRMEM Facility, The University of British Columbia, Vancouver, British Columbia Canada; 4 Department of Microbial Pathogenesis, School of Dentistry, University of Maryland, Baltimore, MD USA; 5 Institute of Marine and Environmental Technology, Baltimore, MD USA; 6 Department of Molecular Biology and Biochemistry, Simon Fraser University, Burnaby, British Columbia Canada; 7 Division of Infectious Diseases, School of Medicine, University of California, San Francisco, San Francisco, CA USA

**Keywords:** Cryoelectron microscopy, Antimicrobial resistance, Enzyme mechanisms, Proteolysis

## Abstract

Broad-spectrum β-lactam antibiotic resistance in *Staphylococcus aureus* is a global healthcare burden^1,2^. In clinical strains, resistance is largely controlled by BlaR1^3^, a receptor that senses β-lactams through the acylation of its sensor domain, inducing transmembrane signalling and activation of the cytoplasmic-facing metalloprotease domain^4^. The metalloprotease domain has a role in BlaI derepression, inducing *blaZ* (β-lactamase PC1) and *mecA* (β-lactam-resistant cell-wall transpeptidase PBP2a) expression^3–7^. Here, overcoming hurdles in isolation, we show that BlaR1 cleaves BlaI directly, as necessary for inactivation, with no requirement for additional components as suggested previously^8^. Cryo-electron microscopy structures of BlaR1—the wild type and an autocleavage-deficient F284A mutant, with or without β-lactam—reveal a domain-swapped dimer that we suggest is critical to the stabilization of the signalling loops within. BlaR1 undergoes spontaneous autocleavage in *cis* between Ser283 and Phe284 and we describe the catalytic mechanism and specificity underlying the self and BlaI cleavage. The structures suggest that allosteric signalling emanates from β-lactam-induced exclusion of the prominent extracellular loop bound competitively in the sensor-domain active site, driving subsequent dynamic motions, including a shift in the sensor towards the membrane and accompanying changes in the zinc metalloprotease domain. We propose that this enhances the expulsion of autocleaved products from the active site, shifting the equilibrium to a state that is permissive of efficient BlaI cleavage. Collectively, this study provides a structure of a two-component signalling receptor that mediates action—in this case, antibiotic resistance—through the direct cleavage of a repressor.

## Abstract (web summary)

Cryo-electron microscopy structures of *Staphylococcus aureus* BlaR1 reveal dynamic signalling states regulating broad spectrum β-lactam antibiotic resistance through cleavage of the transcriptional repressor BlaI and induced expression of the β-lactamase *blaZ* and the β-lactam-resistant cell-wall transpeptidase *mecA*.

## Main

*Staphylococcus aureus* underlies one of the top three most deadly drug-resistant bacterial infections worldwide^2^. Strains of methicillin-resistant *S. aureus* (MRSA) are particularly problematic owing to their prevalence in nosocomial and community-acquired infections, increased rates of morbidity and death, as well as elevated healthcare costs^1^. MRSA is notorious for its ability to acquire resistance to not only β-lactams but also many other classes of antibiotics^1,9^. The drugs typically used to treat MRSA infections, such as vancomycin, are less effective, more toxic and are subject to established and growing resistance mechanisms as evidenced in vancomycin-resistant *S. aureus* strains^9–11^. Despite some progress in the development of new therapeutics^1^, *S. aureus* has recently been estimated to cause more than a quarter of all mortality attributed to antimicrobial resistance in economically developed countries^2^, demonstrating that improved treatment options are urgently required. A clearer understanding of resistance in this pathogen could facilitate the development of new cocktail therapies to preserve β-lactam action.

**Fig. 1:**
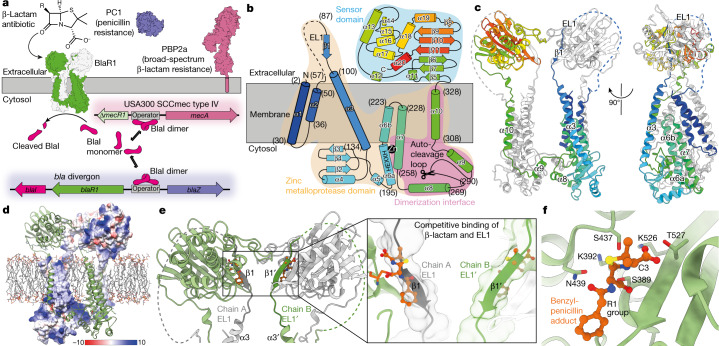
The cryo-EM structure of *S. aureus* BlaR1. **a**, The *bla* pathway controlling β-lactam antibiotic resistance in *S. aureus*. Previously published models for PC1 (Protein Data Bank (PDB): 3BLM) and PBP2a (PDB: 1MWS) were used to generate the figure. **b**, Topology of BlaR1. The zinc metalloprotease and sensor domains are highlighted, as well as the dimerization interface. Residue numbers are indicated in parentheses. **c**, Ribbon representation of the BlaR1 dimer with the monomers shown in either sequence (N to C) rainbow or white. **d**, Dimeric BlaR1 embedded in a lipid bilayer (Methods). Each protomer of the domain-swapped dimer is shown either as a coulombic electrostatic surface (kcal (mol e^−^)^−^^1^ at 298 K) or a green ribbon. Positively charged residues (Arg, Lys, His) proximal to membrane are shown as sticks. SXXK (sensor) and HEXXH (protease) motif residues are shown in ball and stick representation. **e**, Ribbon model of the BlaR1 dimer coloured white for chain A and light green for chain B. BlaR1 EL1 is shown in dark grey and dark green for chains A and B, respectively. A superposed benzylpenicillin molecule is shown in orange from an aligned X-ray structure of the BlaR1 sensor domain (PDB: 1XA7). Inset: EL1 as both a ribbon and a transparent solvent-excluded surface, demonstrating that the modelled position of EL1 occupies the β-lactam-binding cleft and would be competitive with the superposed benzylpenicillin antibiotic. **f**, X-ray structure of the *S. aureus* BlaR1 sensor-domain active site with covalently bound benzylpenicillin (PDB: 1XA7). Residues of key catalytic motifs are shown as sticks.

**Extended Data Fig. 1:**
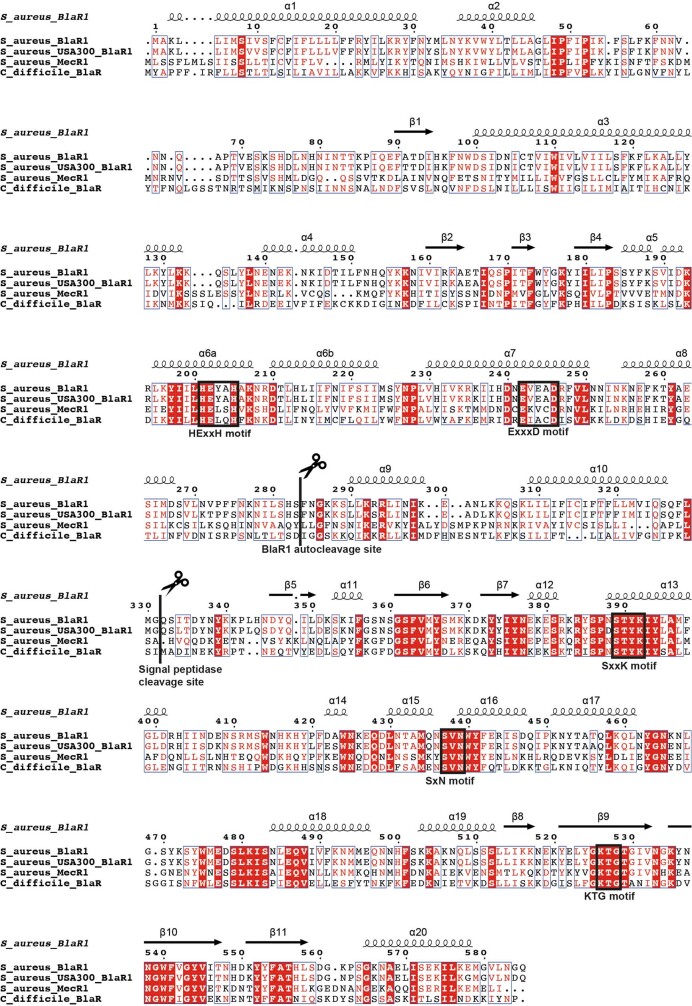
Amino acid sequence alignment of *S. aureus* BlaR1 homologues: *S. aureus* BlaR1 (UniProt ID: Q00419), BlaR1 from MRSA strain USA300 (Refseq ID: WP_001096381.1), *S. aureus* MecR1 (UniProt ID: P0A0B1), and *Clostridioides difficile* BlaR (UniProt ID: Q188R3). *S. aureus* BlaR1 secondary structure is indicated above the sequence alignment.

Inducible β-lactam resistance in *S. aureus* is mediated by the homologous *bla* or *mec* divergons. These encode regulatory systems that sense environmental β-lactams through a membrane-embedded sensor–inducer (BlaR1/MecR1; 35% sequence identity; Extended Data Fig. 1) and induce cleavage of a cytoplasmic transcriptional repressor (BlaI/MecI; 61% sequence identity)^7^. This results in the expression of *blaZ* and *mecA*, which encode β-lactamase PC1^5^ (conferring resistance to penicillin antibiotics) and the altered penicillin-binding protein (enzymes involved in cell-wall synthesis) PBP2a (conferring insensitivity to almost all other β-lactams)^6,12^, respectively (Fig. 1a). The presence of *mec*, embedded in a mobile genetic element called the Staphylococcal cassette chromosome (SCC*mec*), and the production of PBP2a are hallmarks of MRSA^13^. However, there is a complex interplay between divergons. BlaR1–BlaI can control *mecA* expression^3,14,15^ (Fig. 1a), notably inducing PBP2a production in response to β-lactam in minutes compared with hours with MecI–MecR1, which can leave the bacterium susceptible to drug treatment^16^. Accordingly, many SCC*mec* types lack functional MecR1 and/or MecI^17^. One of the predominant MRSA strains in the United States is clonal complex 8 USA300 in which *mecA* is located on a type IV SCC*mec* cassette with a truncated *mecR1* and a deleted *mecI*^17,18^ with expression under the control of plasmid-encoded BlaR1 and BlaI. More generally, type IV SCC*mec* or its closely related subtypes are frequently encountered owing to their presence in epidemic community MRSA strains worldwide. Thus, BlaR1 and BlaI are critically important in determining broad-spectrum antibiotic resistance in many MRSA strains.

BlaR1 and MecR1 are membrane proteins with an N-terminal zinc metalloprotease domain and a C-terminal extracellular β-lactam-binding sensor domain. Isolated structures of the β-lactam-binding sensor domain in complex with antibiotics have been determined for *S. aureus* BlaR1 and MecR1, showing near-identical architectures (Cα root mean squared deviation of around 2.9 Å) that are related to the class D serine β-lactamases^15^. No structures of the transmembrane and proteolytic regions have been determined and the signal transduction events linking β-lactam binding and BlaI cleavage on opposing sides of the membrane remain unclear^19–21^. To address these unknowns, we present structures of full-length *S. aureus* BlaR1. On the basis of conformational differences with and without the β-lactam ampicillin, we propose a mechanism of *bla* regulation that defines BlaR1 as a two-component signalling receptor. These insights provide a molecular foundation for the development of therapeutics for both MRSA and homologous pathways in other drug-resistant pathogens, including *Mycobacterium tuberculosis*^22^ and *Clostridioides difficile*^23^.

## Cryo-EM analysis of BlaR1

**Extended Data Fig. 2:**
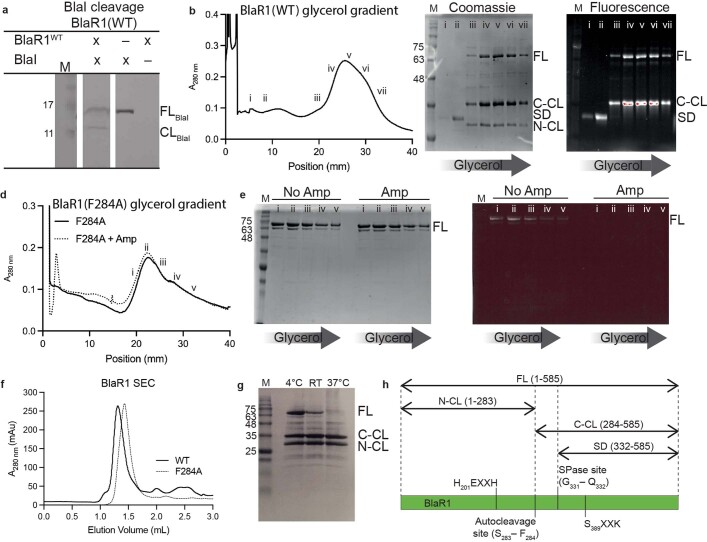
Characterization of *S. aureus* BlaR1 by glycerol gradient centrifugation, SDS-PAGE and size exclusion chromatography. **a**, WT BlaR1 in membranes cleaves T7-tagged BlaI as shown by an anti-T7-tag antibody probed Western blot. Representative gel shown from three independent replicates. **b**, Glycerol gradient chromatogram of BlaR1(WT). **c**, Coomassie stained (left) and fluorescent (BOCILLIN FL) imaged (right) SDS-PAGE gel of BlaR1(WT) fractions from a glycerol gradient. Samples were incubated with BOCILLIN FL, a fluorescently labelled β-lactam antibiotic, prior to SDS-PAGE. BlaR1(WT) undergoes spontaneous autocleavage. BlaR1(WT) full-length (FL), the BlaR1(WT) C-terminal autocleavage product including residues 284-585 (C-CL), the BlaR1(WT) N-terminal autocleavage product including residues 1-283 (N-CL) and BlaR1(WT) sensor domain including residues 332-585 (SD) bands are indicated. Representative gel shown from multiple (>10) independent purifications. **d**, Glycerol gradient chromatogram of BlaR1(F284A) with and without ampicillin. **e**, Coomassie stained (left) and fluorescent (BOCILLIN FL) imaged (right) SDS-PAGE gel of BlaR1(F284A) fractions from the glycerol gradients with and without ampicillin as indicated. Acylation with ampicillin confirmed by inhibition of BOCILLIN binding. Representative gel shown from multiple (>5) independent purifications. **f**, Size exclusion chromatography using a Superdex 200, 5/150 column shows BlaR1(WT) and BlaR1(F284A) elute as single peaks. The later elution profile of the BlaR1(F284A) sample suggests it may form a more compact dimer. **g**, Purified BlaR1(WT) undergoes spontaneous cleavage. Samples were loaded on an SDS-PAGE gel following incubation for 1 day following purification at the temperature indicated. Representative gel shown from two independent replicates. **h**, Schematic of the BlaR1 cleavage products. Molecular weights of the protein ladder components are indicated in kDa for all gels. Gels in panels C and F were scanned for fluorescence using a Chemi Genius 2 Bio Imaging System (Syngene). For gel source data, see Supplementary Fig. 1.

To overcome the historical challenge of obtaining adequate quantities of active BlaR1^21,24^, we used the nisin-controlled gene expression system in *Lactococcus lactis*^25^. Membranes containing expressed wild-type BlaR1 (BlaR1(WT)) were able to cleave BlaI, suggesting that the zinc metalloprotease domain is active (Extended Data Fig. 2a), as shown previously in *Escherichia coli* membranes containing expressed *S. aureus* BlaR1^26^. Purification of BlaR1 was monitored by the covalently bound fluorescent β-lactam BOCILLIN FL^27^. As in *S. aureus* and *E. coli*^28^, BlaR1(WT) expressed in *L. lactis* undergoes spontaneous autocleavage (Extended Data Fig. 2c) between residues Ser283 and Phe284 (confirmed by Edman N-terminal sequencing), which was mitigated by mutation of Phe284 in the P1′ position to alanine (BlaR1(F284A)) (Extended Data Fig. 2e). Detergent-solubilized BlaR1(WT) and BlaR1(F284A) behave like oligomers (Extended Data Fig. 2f) and rate-zonal ultracentrifugation was used to enrich the sample for the intact oligomeric species (Extended Data Fig. 2b–e).

**Extended Data Fig. 3:**
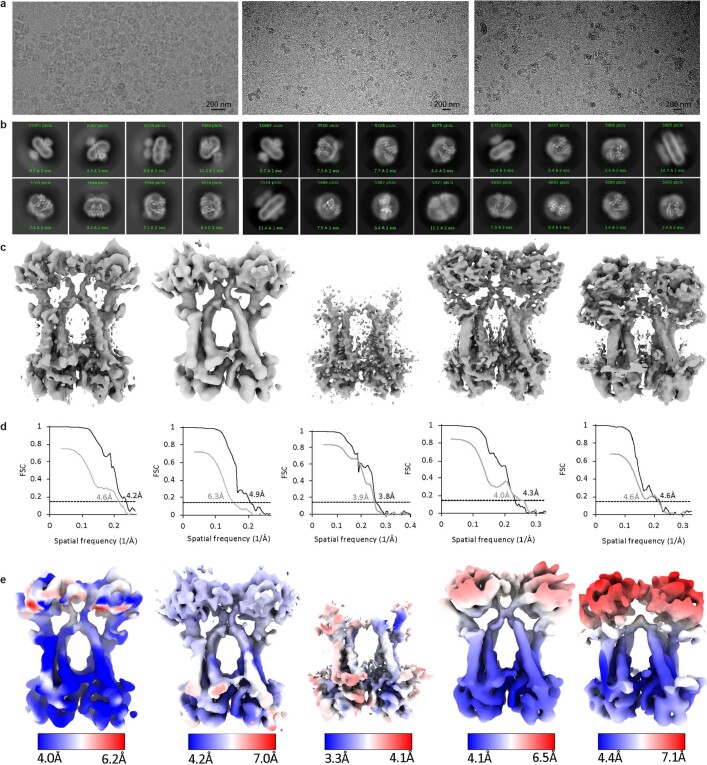
Single particle cryo-EM data processing of BlaR1. **a**, Representative micrographs for BlaR1(WT), BlaR1(F284A) and BlaR1(F284A)-ampicillin, respectively. **b**, Representative 2D classes for BlaR1(WT), BlaR1(F284A) and BlaR1(F284A)-ampicillin, respectively. **c**, 3D reconstructions BlaR1(WT) C2, BlaR1(WT) C1, BlaR1(WT) TM, BlaR1(F284A) and BlaR1(F284A)-ampicillin, respectively. **d**, Gold-standard half-map FSC curves as in C (black line) and model-map FSC curves (grey line). Dashed line at 0.143 FSC. **e**, As **c** coloured according to local resolution.

**Extended Data Fig. 4:**
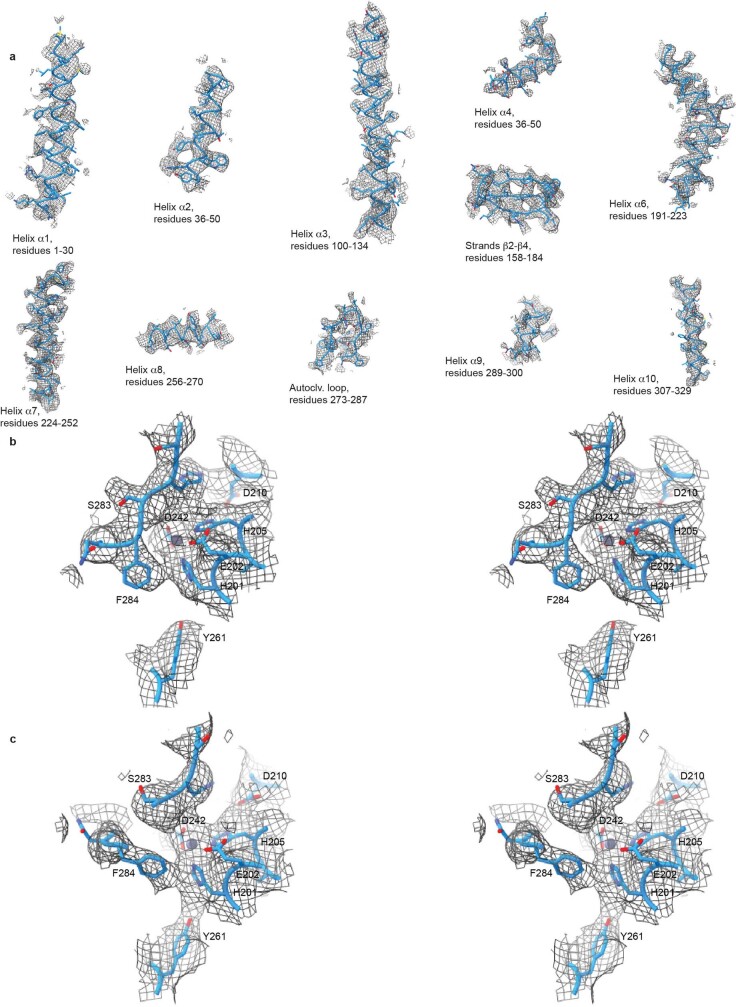
Example cryo-EM density displayed for the BlaR1(WT) map. **a**, Density for BlaR1(WT) model secondary structure elements shown in mesh. **b** and **c**, Stereo views of active site with an intact autocleavage loop (**b**) or cleaved autocleavage loop (**c**).

**Extended Data Fig. 5:**
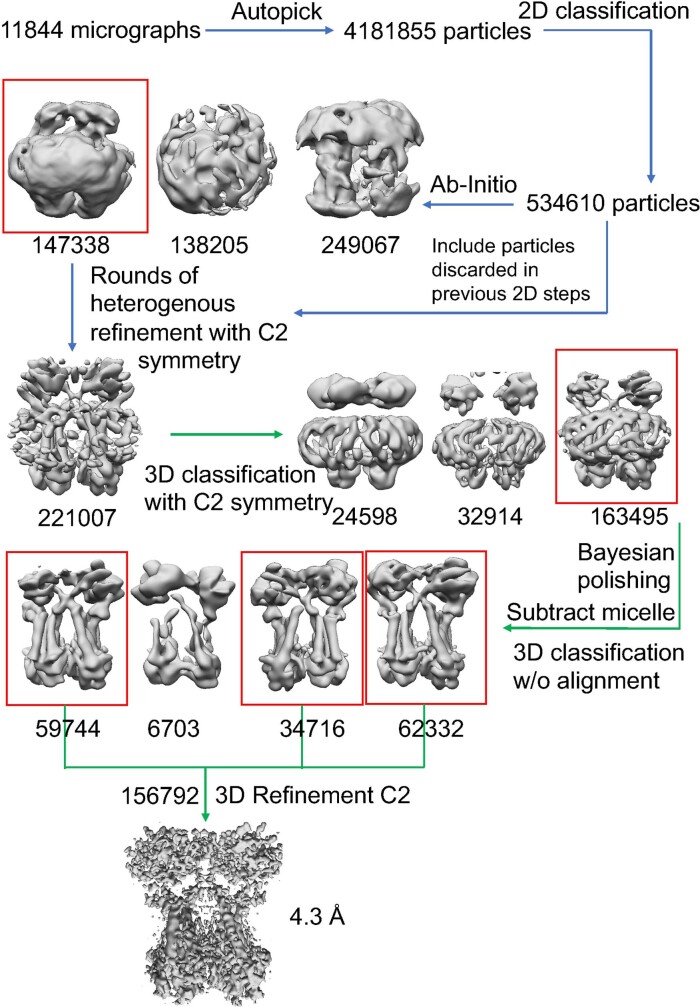
Example single particle cryo-EM data processing strategy of ligand-free BlaR1. Blue and green arrows correspond to processing in RELION^64^ and cryoSPARC^65^, respectively.

The structures of BlaR1(WT) and BlaR1(F284A) (the latter alone and in complex with ampicillin (Extended Data Fig. 2e)) were determined using cryo-electron microscopy (cryo-EM) to nominal resolutions of 3.8–4.6 Å (Fig. 1, Extended Data Figs. 3–5 and Extended Data Table 1). BlaR1 was consistently observed in a dimeric form (Extended Data Fig. 3b,c) with two-fold symmetry averaging applied in the overall reconstructions for each sample. More structural heterogeneity was detected in the BlaR1(WT) dataset compared with the uncleavable mutant BlaR1(F284A), suggesting enhanced dynamic motions. An asymmetric homodimer class was identified, resulting in a reconstruction of the previously uncharacterized BlaR1(WT) transmembrane and zinc metalloprotease region to 3.8 Å with a local resolution extending to about 3.3 Å (Extended Data Fig. 3e).

## BlaR1 forms a domain-swapped dimer

**Fig. 2:**
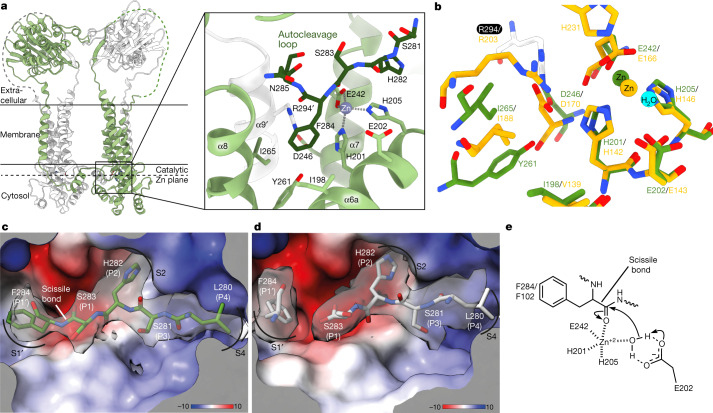
The zinc metalloprotease active site of BlaR1 and binding of the autocleavage loop substrate and product. **a**, The BlaR1 dimer is shown as a ribbon and is coloured by monomer. The position of the bound catalytic zinc (dashed line) relative to the membrane leaflet (solid line) is indicated. The box out shows the labelled zinc metalloprotease active-site residues, highlighting the intermolecular composition (residues are shown in stick with CPK colouring, with carbons in light green, except for those of the domain-swapped contributing residue Arg294, which are shown in white). Autocleavage loop residues are shown in darker green. **b**, Alignment of BlaR1 zinc metalloprotease active-site residues (depicted as described in **a**) with thermolysin (PDB: 2TLX), shown in CPK with gold carbons. The proposed thermolysin catalytic water is represented as a cyan sphere. **c**,**d**, Electrostatic surface representation (kcal (mol e^−^)^−1^ at 298 K) of the BlaR1(WT) active-site cleft with intact (**c**) and cleaved (**d**) autocleavage loop residues, shown in CPK colouring with green and white carbons, respectively. The cryo-EM map is shown as a transparent grey surface. See also Extended Data Fig. 4b,c. **e**, A 2D representation of the proposed steps leading to the formation of a oxyanion tetrahedral transition state in the zinc metalloprotease active site.

**Extended Data Fig 6:**
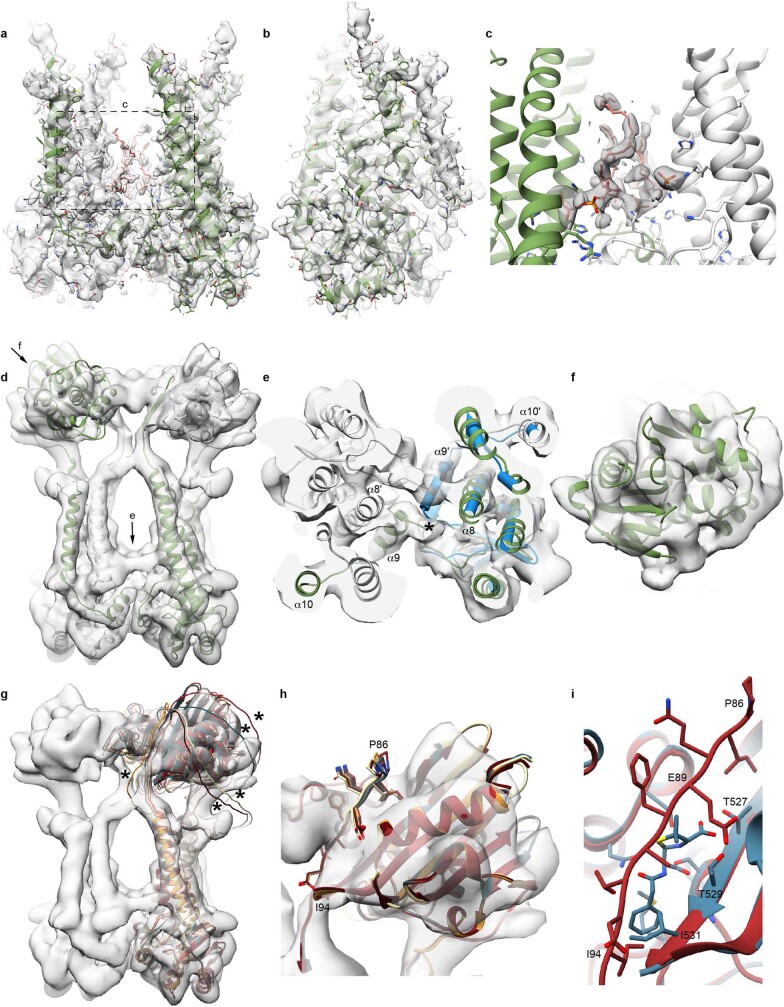
Cryo-EM model building for BlaR1. **a**, Model of the BlaR1 TM region built using the BlaR(WT) TM map at 3.8 Å resolution. **b**, Side view as in **a**. **c**, Boxed region in **a** showing two phosphatidylglycerol (16:0/16:0) lipids and three phosphates (presumably corresponding to lipid headgroups) modelled in the central chamber between TM regions. Positively charged residues (arginine, lysine, histidine) are shown as sticks. **d**, Model of BlaR1 including sensor domain built using the BlaR1(WT) C2 reconstruction at 4.2 Å resolution. Views in **e** and **f** indicated. **e**, View indicated in **d** showing domain swap mediated dimerization. The domain swap occurs between helices α8 and α9 (*). An AlphaFold2 monomeric model is shown as blue cylinders. **f**, Sensor domain view as indicated in **d** showing fit of X-ray structure (1XA7) in density. **g**, Five AlphaFold2 BlaR1 monomeric models docked in the BlaR1(WT) C2 reconstruction based on the TM region. The different EL1 loop predictions (residues 57-85; low confidence) are indicated with *. **h**, AlphaFold2 models fit in map based on sensor domain. The C-terminal residues 86-96 of EL1 are consistently predicted bound in the sensor domain β-lactam binding groove with increased confidence (pLDDT of 60-75; >70 indicates a confident prediction). **i**, Benzylpenicillin bound to sensor domain (1XA7) overlaid with representative AlphaFold2 model.

Notably, BlaR1 is observed as an extensive and intimate dimer mediated by an unambiguous domain swap (Fig. 1c,d, Extended Data Fig. 6e and Supplementary Video 1). The N-terminal zinc metalloprotease domain is well-ordered and includes four full transmembrane α-helices, a re-entrant loop (often implicated in meditating protein channel specificity, transport or signalling functions)^29^ and a cytosolic zinc metalloprotease active site defined by the characteristic H_201_EXXH and E_242_XXXD gluzincin signature motifs^30^ (Figs. 1b and  2 and Extended Data Fig. 1). The active site projects towards the cytosolic face, allowing a point of access for BlaI binding, while the soluble C-terminal β-lactam-sensing domain protrudes from the opposing extramembranal face of the bilayer (Figs. 1 and 2). The N terminus is also oriented to the extracellular side of the membrane leading to an N_out_, C_out_ topology (Fig. 1b,c), which is rarely observed in bacterial membrane proteins^31,32^.

The domain swap involves the cytosolic-facing helix α9 of the zinc metalloprotease domain and helix α10, the terminal transmembrane helix, resulting in an extensive interface with a buried surface area of about 3,165 Å^2^. Within the zinc metalloprotease domain, additional interface interactions involve helices α8 (same chain) and α9′ (domain swapped), which are oriented almost parallel to the membrane, and helix α7, the C-terminal re-entrant helix that houses the H_201_EXXH motif. Dimerization defines a central cavity, which in the native membrane is presumably filled with lipids (Fig. 1d). Densities consistent with phosphatidylglycerol headgroups are observed lining the electropositive surface formed at the base of the cavity. Additional diffuse density for fatty acid chains enabled modelling of phosphatidylglycerol(16:0/16:0), a common lipid found in *L. lactis* and *S. aureus*^33–36^ (Extended Data Fig. 6a–c).

The loop connecting helices α8 and swapped α9, housing the scissile bond between residues Ser283 and Phe284 (residues 271 to 289; here termed the autocleavage loop), enters the zinc metalloprotease active site of its own chain, directly demonstrating that autocleavage occurs in *cis* (Fig. 2a). The swapped position of α9 effectively lengthens the distance between the autocleavage loop tether points and creates a stabilizing set of hydrophobic core contacts at the dimeric interface involving a number of conserved side chains. In the BlaR1(F284A) mutant structures, the autocleavage loop is intact; however, in the BlaR1(WT) structure, we detected a mixture of uncleaved and cleaved forms, the latter with the central P1′ Phe284 slightly shifted in position (Fig. 2c,d, Extended Data Fig. 4b,c and Supplementary Video 2). This observation is consistent with the presence of both cleaved and uncleaved BlaR1(WT) observed using SDS–PAGE (Extended Data Fig. 2c). We propose that dimerization results in encapsulation of the autocleavage loops within the lumen of the dimer, potentially providing enhanced stability of these dynamic signalling regions to unbridled conformational freedom and/or random hydrolysis.

**Fig. 3:**
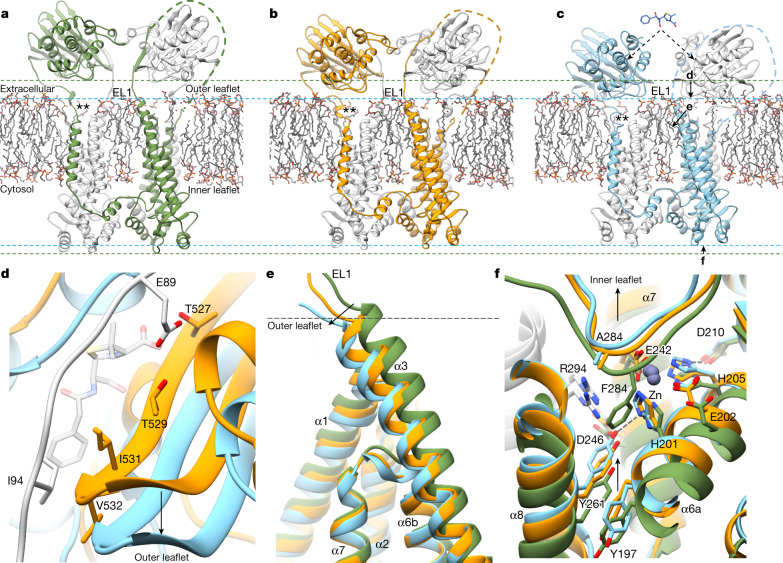
Conformational changes after β-lactam binding to the extracellular sensor domain, resulting in EL1 displacement, sensor reorientation, and transmembrane and cytosolic-facing metalloprotease domain rearrangements. **a**–**c**, Structures of dimeric BlaR1(WT) (**a**; green/white ribbon), BlaR1(F284A) (**b**; orange/white ribbon) and BlaR1(F284A)–ampicillin (**c**; cyan/white ribbon). The dashed lines highlight the extreme positions of the sensor (extracellular) or zinc metalloprotease (cytosol) domains with respect to the membrane and are coloured according to the BlaR1(WT) or BlaR1(F284A)–ampicillin structures. The labelled arrows in **c** highlight the β-lactam-induced movement towards the outer and inner leaflets as expanded on in **d**–**f**. Unresolved EL1 residues 56–86 are indicated by dashed lines. The double asterisk indicates the location of the determined signal peptidase cleavage site Gly331. **d**, Benzylpenicillin from PDB 1XA7 (transparent grey) superposed onto **b** (BlaR1(F284A), orange), showing the structural overlap with the modelled EL1 loop (white). β-Lactam binding as described in **c** (BlaR1(F284A)–ampicillin, cyan) induces a shift in the sensor domain towards the outer leaflet of the membrane, we propose as a result of competitive displacement of EL1 from the sensor-domain active site. **e**, β-Lactam-induced motions of transmembrane helix α3, directly downstream of EL1, coloured as described in **a**–**c**. The distance between the sensor domain and the membrane correlates with increased displacement of the N-terminal region of α3. **f**, Superposed active-site structures from BlaR1 structures in **a**–**c**. The F284A mutation results in an unoccupied S1′ pocket and associated movement of Tyr261 on helix α8 towards His201 of the HEXXH motif in **b** (orange) and **c** (cyan; dashed line). Supporting data are provided in Extended Data Fig. 7. Superpositions in **d**–**f** are based on structural alignment of the re-entrant helices containing the catalytic HEXXH…E motif.

**Extended Data Fig. 7:**
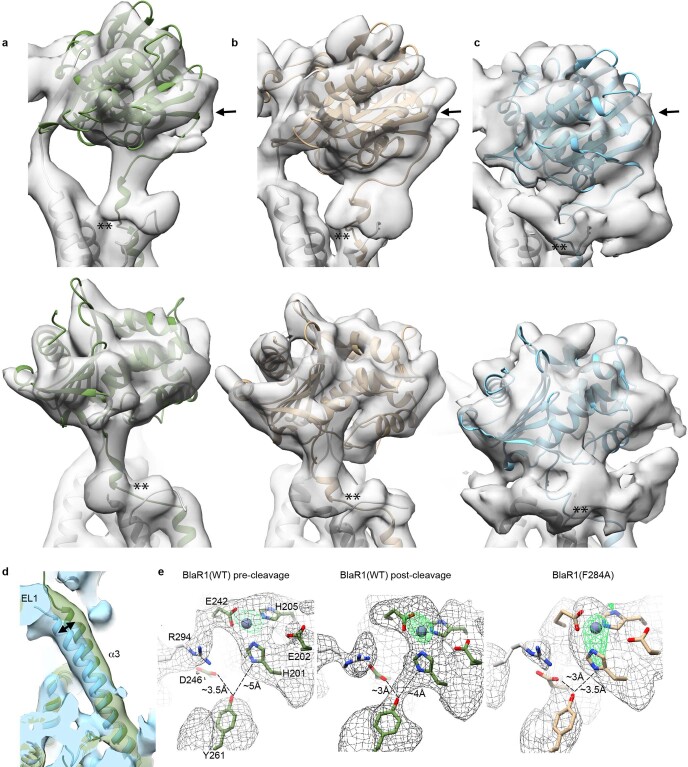
Conformational changes associated with autocleavage and β-lactam binding. **a**–**c**, Sensor domains modelled into BlaR1(WT) C2 (**a**), BlaR1(F284A) (**b**) and BlaR1(F284A)-ampicillin (**c**) reconstructions. Top front view, bottom side view (indicated with arrow in top panel). Putative signal peptidase cleavage site between Gly330 and Gln331 indicated (**). **d**, Helix α3 position in BlaR1(WT) (green) and BlaR1(F284A)-ampicillin (cyan) representing the two conformational extremes. For comparison of conformational changes, models were first superposed based on residues 200-250 (HEXXH…E motif including re-entrant helix) and reconstructions superposed on these models. **e**, Active site density showing approximate distance between Tyr261 and His201/Asp246. Tyr261 moves closer in the post-cleavage state of the BlaR1(WT) TM reconstruction and in BlaR1(F284A) where the phenylalanine to alanine mutation results in an empty S1′ pocket. Cryo-EM map shown as mesh contoured at levels to best display protein (grey) and catalytic zinc position (green).

The extracellular BlaR1 sensor domain was less-well resolved, suggesting a more dynamic relative orientation. The reconstructions of BlaR1(WT) and BlaR1(F284A) without ampicillin provided sufficient secondary structure features (local resolution 6–6.5 Å) to enable confident placement of the sensor-domain X-ray crystal structure^37^ (Extended Data Figs. 6d,f and  7a–c), which is oriented with the β-lactam-binding grooves facing each other (Fig. 1c–e). In the BlaR1(WT) dataset, the sensor domains were observed to be dynamic and are able to adopt different orientations with respect to each other and the transmembrane domains (Fig. 3a, Extended Data Fig. 7 and Supplementary Video 3). A comparison with BlaR1(F284A) shows that the sensor domains are positioned at different distances to the transmembrane domain in order of BlaR1(WT), BlaR1(F284A) and BlaR1(F284A)–ampicillin (Fig. 3a–c and Extended Data Fig. 7a–c).

The extracellular loop (EL1; residues 56–96), which has long been hypothesized to be involved in BlaR1 signalling^19,20^, was observed to protrude from transmembrane helices α2 and α3 at the outer leaflet into the region adjacent to the sensor domain of the neighbouring monomer as a result of the domain swap (Fig. 1c–e). Like the sensor domains, the EL1 loops are less-well resolved, consistent with a more dynamic nature. EL1 was modelled using AlphaFold2^38^ as a starting template and optimized using Rosetta density-guided refinement^39^ (Fig. 1e, Extended Data Fig. 6g–i and Supplementary Video 1). Conserved residues Ile87–Ile94 were specifically observed occupying the β-lactam-binding groove in *trans*. Notably, acidic Glu89 (consensus Asp) and hydrophobic Ile94 (consensus Val) occupy a similar space to the C3 β-lactam carboxylate and hydrophobic R1 groups in structures of isolated acylated sensor domains (Fig. 1e,f and Extended Data Fig. 6i). The β-lactam carboxylate forms a near universal favourable electrostatic interaction with the adjacent electropositive KTG motif (Lys526 in BlaR1) and the R1 group forms extensive van der Waals interactions with the edge strand in the active site (BlaR1 residues Gly530–Val532). In support of these competitive binding interactions, a peptide with the sequence of EL1 has previously been found to interact with the sensor domain in solution^19,20,40^. It is clear that acylation of the sensor-domain active site serine by β-lactam antibiotics will displace the EL1 loop from the active site, a structural hallmark of initiation of downstream signalling events now illuminated here.

## The protease active site and cleavage mechanism

Helices α6, α7 and β-strand β3 together take on a thermolysin-like fold (the prototypical gluzincin), protruding from a transmembrane scaffold, with analogous positioning of several key active site residues (Fig. 2b). Readily identified by localization of the gluzincin catalytic metalloprotease HEXXH…E sequence motif^41^, BlaR1 helix α6a here positions His201 and His205, along with Glu242 from neighbouring helix α7, to coordinate the clearly observed catalytic zinc in the expected tetrahedral manner (Fig. 2a and Extended Data Figs. 4b,c and 7e) and present at a 1:1 molar ratio with BlaR1 (Supplemental Table 1). Helices α8 (specifically Ile265 in the S1′ subsite and Tyr261 approaching His201) and α9′ (Arg294′, which forms a stabilizing interchain salt bridge with Asp246) both contribute conserved residues to the active site, suggesting that their precise position is important for function and supporting the observed domain-swapped dimer^42^ (Fig. 2a,b).

**Extended Data Fig. 8:**
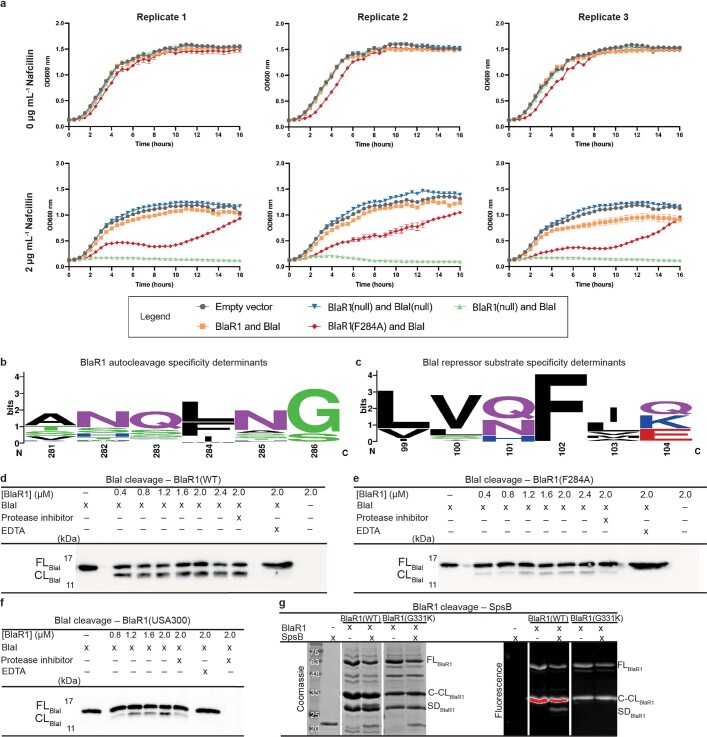
Growth curves for MRSA USA300 in the absence and presence of 2 µg ml^−1^ nafcillin and BlaR1 and BlaI cleavage. **a**, The F284A mutation in BlaR1 resulted in an attenuated bacterial survival in presence of nafcillin. Growth assay of SF8300ermS containing pTXΔ with variants of *blaR1* and *blaI* in absence of antibiotics showed similar growth rates among the selected strains. Growth assay of SF8300ermS containing pTXΔ with variants of *blaR1* and *blaI* in presence of 2 µg ml^−1^ nafcillin showed that the F284A functional mutation in *blaR1* (red diamonds) caused an obvious growth defect when compared to the wild type (yellow squares). For each condition three technical replicates and three biological replicates were performed. Data from each biological replicate is graphed separately on each row with growth under 0 µg ml^−1^ nafcillin shown in the left column and growth under 2 µg ml^−1^ shown in the right column. The mean and standard deviation of the technical replicates (n = 3) are shown for each treatment. **b**, Sequence conservation plot generated with the WebLogo server (https://weblogo.berkeley.edu/) of residues flanking the BlaR1 autocleavage site between residues Ser283 (P1) and Phe284 (P1′). **c**, Sequence conservation plot of residues flanking the BlaI cleavage site between residues Asn101 (P1) and Phe102 (P1′). **d**, BlaR1(WT), **e**, BlaR1(F284A) and **f**, BlaR1(USA300) mediate cleavage of BlaI as shown by Western blot. A 2× working concentration of cOmplete protease inhibitor cocktail (Roche) did not prevent BlaI cleavage. However, cleavage was abrogated with the addition of 100 mM EDTA. For panels c, d and e full-length (FL_BlaI_) and cleaved (CL_BlaI_) T7-tagged BlaI bands are indicated. BlaI cleavage experiments in **d** – **f** were carried out a minimum of three times with similar results, representative blots shown. **g**, *S. aureus* signal peptidase SpsB mediates cleavage of the BlaR1(WT) sensor domain as demonstrated by SDS-PAGE stained with Coomassie and BOCILLIN FL fluorescently labelled BlaR1 sensor domain. BlaR1 full-length (FL), the BlaR1 C-terminal autocleavage product including residues 284-585 (C-CL), the BlaR1 N-terminal autocleavage product including residues 1-283 (N-CL) and BlaR1WT sensor domain including residues 332-585 (SD_BlaR1_) bands are indicated. The BlaR1 G331K (BlaR1(G331K)) mutation in the P1 position of the SpsB cleavage site protects against SpsB mediated cleavage. We predict SpsB mediated cleavage is important for BlaR1 turnover in the absence of β-lactam antibiotic. The molecular weights of selected bands of the protein ladder are indicated in kDa for panels C-F. SpsB cleavage was carried out two times with similar results. For gel source data, see Supplementary Fig. 1.

The initial zinc metalloprotease substrate, the autocleavage loop captured here in uncleaved and cleaved states (Fig. 2c,d), provides insights into the molecular details of substrate specificity. The P4–P1′ residues of the autocleavage loop (Leu280, Ser281, His282, Ser283, Phe284) adopt the typical β-strand conformation of protease substrates^43^, which runs anti-parallel to β3 of the gluzincin domain β-sheet analogous to substrate binding in thermolysin related proteases (Fig. 2). In addition to main-chain β-strand hydrogen bonds between Thr172 and His282, the substrate is anchored by hydrophobic interactions of Leu280 into the S4 pocket at one end and P1′ Phe284 inserting into the S1′ subsite at the other, with the intervening P2 His282 within the S2 pocket the other important specificity determinant. The well-defined S1′ subsite, typically the major determinant for substrate specificity in endolytic metalloproteases^44^, has a large hydrophobic cavity that is suited for the binding of the aromatic phenylalanine side chain of P1′ Phe284 (Fig. 2c,d). Sequence conservation at this position shows that there is a preference for a bulky hydrophobic amino acid (Extended Data Fig. 8b). By contrast, phenylalanine at the P1′ of the BlaI repressor cleavage sequence is almost invariant (Extended Data Fig. 8c), hinting at a tighter affinity that may be important in displacing autocleaved products from the BlaR1 metalloprotease active site.

In the WT structure, one chain shows clear separation of Ser283(P1) and Phe284(P1′) supporting a post-autocleavage state (Fig. 2c,d and Extended Data Fig. 4b,c). By contrast, the density in the alternate chain is more uninterrupted, suggesting a pre-cleavage state, consistent with a population of both uncleaved and cleaved protein in the sample used for cryo-EM. The autocleavage loop is in the approximate position for cleavage, with the P1 Ser283 carbonyl orientated favourably towards the catalytic zinc, which canonically acts to polarize the scissile bond, promoting its electrophilicity and subsequent nucleophilic attack by an activated water to promote cleavage. Although we observed the same binding orientation in the autocleavage-deficient BlaR1(F284A) structure, the P1′ alanine mutant leads to the loss of the close van der Waals-driven socketing of the aromatic phenylalanine side chain into the S1′ pocket, preventing productive coordination of the carbonyl oxygen to the zinc. Although the hydrolytic water was not observed at this resolution, by comparison with thermolysin, we expect that Glu202 in BlaR1 coordinates the catalytic water and provides general base activation to the nucleophilic form for attack on the P1 carbonyl and subsequent hydrolysis (Fig. 2b,e).

Notably, both the intact and cleaved autocleavage loop forms occupy the catalytic cleft such that their presence is not sterically compatible with the binding and cleavage of BlaI. Thus, our structures show that movement of the autocleavage loop out of the active site, either in an intact or cleaved state, is needed for cleavage of BlaI to proceed. We suggest again a role for the observed dimerization in encompassing and stabilizing these loops in both forms at the dimeric interface, minimizing their susceptibility to random proteolysis that would presumably be enhanced in the more exposed, even less stable monomeric forms.

## BlaR1 autocleavage is spontaneous

The role of autocleavage in the regulation of BlaR1 catalytic activity and in turn the BlaR1 divergon has been an important focus^4,26,28^. Here, BlaR1(WT) undergoes spontaneous autocleavage (Extended Data Fig. 2c), consistent with previous reports^4,26,28^. Autocleavage is time and temperature dependent with substantial albeit incomplete cleavage at 4 °C, which was used for cryo-EM sample preparation, and near-complete fragmentation overnight at room temperature or 37 °C (Extended Data Fig. 2g).

To further investigate the role of autocleavage, we compared the growth of BlaR1(WT) and BlaR1(F284A) in the common clinical variant USA300 MRSA strain SF8300, in which *mecA* expression is under control of a *blaI*-*blaR1* plasmid, in the presence of the β-lactam antibiotic nafcillin (Extended Data Fig. 8a). In the absence of antibiotic, all of the strains displayed a similar growth pattern. Nafcillin treatment fully inhibited the growth of the strain containing *blaR1(null)-blaI*, presumably due to its inability to derepress BlaI. The strains containing an empty vector or a non-functional *blaR1(null)-blaI(null)* construct did not produce growth inhibition, suggesting constitutive *mecA* expression in absence of a functional BlaI repressor. The strain containing BlaR1(F284A) (*blaR1(F284A)-blaI*) displayed intermediate growth attenuation in presence of nafcillin compared with either wild-type BlaR1 (*blaR1-blaI*) or the functionally defective BlaR1 (null) (*blaR1(null)-blaI*) (Extended Data Fig. 8a), showing that the F284A mutant is partially functional.

We further examined the ability of purified BlaR1—including both the variants used in our structural studies and that used in the growth assay above (BlaR1(USA300))—to directly cleave purified BlaI. BlaR1(WT) and BlaR1(USA300) cleave BlaI in a manner that is completely abrogated by EDTA but not serine/cysteine protease inhibitors, supporting specific metalloprotease activity (Extended Data Fig. 8d–f). Edman N-terminal sequencing confirmed cleavage between BlaI residues Asn101 (P1) and Phe102 (P1′), as previously identified in BlaR1-containing *S. aureus* membranes^4^. Autocleavage-deficient BlaR1(F284A) also shows BlaI cleavage, albeit at a reduced level compared with BlaR1(WT), suggesting that some level of BlaI binding and cleavage is still possible in the presence of an intact autocleavage loop (barring a minor population of autocleaved BlaR1(F284A) that is not detectable in our fluorescence-based gel assay (Extended Data Fig. 2e).

Collectively, these results suggest that the autocleavage event is spontaneous and is not part of the signal transduction mechanism itself, and it does not appear to be an absolute requirement for BlaI cleavage and activation of the *bla* divergon to occur, although it does permit a more efficient response to environmental β-lactams that may be required for resistance in vivo. These data also show that BlaR1 can recruit and cleave BlaI directly with no need for intermediary components, as was proposed previously^8^.

## β-Lactam-induced allosteric changes

β-Lactam binding to the extracellular BlaR1 sensor domain regulates BlaI cleavage by the zinc metalloprotease domain in the cytoplasm. The three structures presented here reveal conformational changes linking the two BlaR1 active sites about 65 Å apart across the intervening membrane. The overall transmembrane and zinc metalloprotease domains are similar across structures suggesting that no major conformational rearrangements are induced on acylation, although subtle motions of select helices were observed that we believe are functionally important. By contrast, we observed that the sensor domain is more dynamic (Fig. 3a). Focused 3D classification shows that the BlaR1(WT) sensor domain samples multiple orientations, although none refine to a high resolution, which indicates a continuous dynamic trajectory. 3D variability analysis^45^ shows a general up/down translation of 4–5 Å relative to the membrane (Supplementary Video 2). The BlaR1(F284A) structure (refined with *C*_2_ symmetry) exhibited less variability and the sensor domains adopt a position slightly closer to the membrane compared with BlaR1(WT) (Fig. 3a–c and Extended Data Fig. 7a–c). However, in the presence of ampicillin, we see a pronounced rigid body shift of about 6 Å to a position immediately abutting the membrane. The magnitude of this shift is consistent with the expected displacement of EL1 induced by the competitive binding of ampicillin (Fig. 3d). Thus, β-lactam binding and the consequent extrusion of EL1 from the active site appears to trigger movement of the sensor domain towards the outer leaflet of the membrane.

Correlated motions are also observed in the transmembrane and zinc metalloprotease domains. Notably, a general compression parallel to the membrane normal was observed in the BlaR1(F284A)–ampicillin structure. Careful superposition of the reconstructions onto the re-entrant helices containing the catalytic HEXXH…E motif clearly demonstrates that structural elements on either side of the membrane are compressed towards each other in the presence of ampicillin (Fig. 3a–c and Extended Data Fig. 7). As above, a key determinant of the observed motions appears to be the steric displacement of EL1 by the shifted sensor domain. EL1 is situated immediately upstream of helix α3, which is observed kinked into the outer leaflet of the membrane as a result of the shift of EL1 (Fig. 3e and Extended Data Fig. 7d). This is coupled to a general movement of the distal region of the zinc metalloprotease domain towards the inner leaflet of the membrane (Fig. 3a–c and Supplementary Video 3).

## Discussion

The structure of *S. aureus* BlaR1 has been unclear since the initial identification of the gene over 30 years ago^3,46^. This paper advances knowledge of BlaR1-mediated antibiotic resistance, providing insights into the mechanism of BlaR1 signal transduction that could facilitate the development of therapeutics to treat MRSA infections.

**Extended Data Fig. 9:**
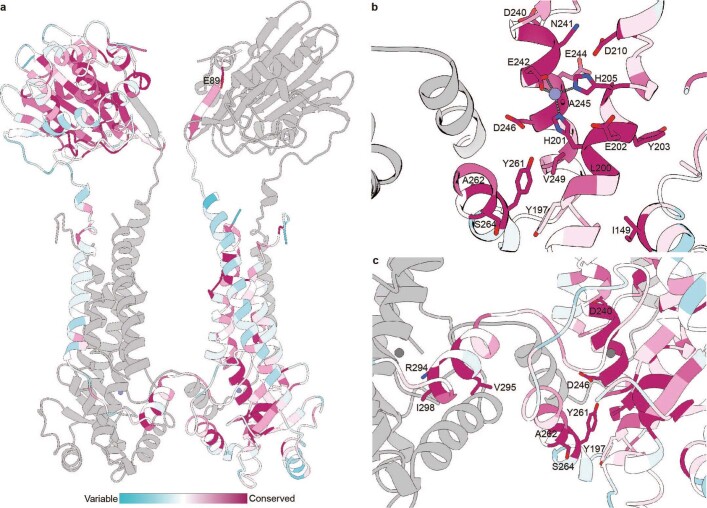
Mapping of BlaR1 sequence conservation. Chain A of the BlaR1 dimer is coloured by sequence conservation^81^ while chain B is coloured grey. Areas where sequence conservation is uncertain are coloured light yellow. **a**, Overall sequence conservation of the modelled BlaR1 residues. Focus in **b**, the BlaR1 zinc metalloprotease active site and in **c**, the BlaR1 domain swap interface with selected conserved residues shown as sticks.

BlaR1 forms a domain-swapped dimer, with the zinc metalloprotease active site accessible from the cytoplasm. The gluzincin fold superposes well with the prototypical soluble representative thermolysin and the most closely related integral membrane zinc metalloproteases with known structures: eukaryotic nuclear membrane ZMPSTE24^47^ and Ste24p^48^. In addition to the conserved H_201_EXXH and E_242_XXXD motifs, the analysis further defines residues that are critical to catalytic function. Asp210 and Tyr261 are near strictly conserved and are positioned to stabilize core catalytic residues His205 or His201, respectively (Fig. 3f and Extended Data Fig. 9). Mutation of either to alanine abolished β-lactamase induction in *B. licheniformis*^49^. Asp246 forms a salt bridge with Arg294′, an interaction that is conserved in other gluzincins; however, in BlaR1, this is located on domain-swapped helix α9′ and forms an intermolecular interaction. In thermolysin, the arginine side chain binds to the substrate P1′ carbonyl^42^. Consistently, Arg294′ projects towards Phe284 of the autocleavage loop in BlaR1. Mutation of Asp246 abolished β-lactamase induction in *B. licheniformis*, whereas double mutation of Arg293 and Arg294 severely diminished activity^49^. We did not observe an equivalent of thermolysin His231, which is proposed to be involved in transition-state binding and stabilization^50^ (Fig. 2b). His231 is conserved in other gluzincins, including ZMPSTE24, in which it is located in a loop connecting helices equivalent to BlaR1 α8 and α9, which in BlaR1 is the autocleavage loop.

**Fig. 4:**
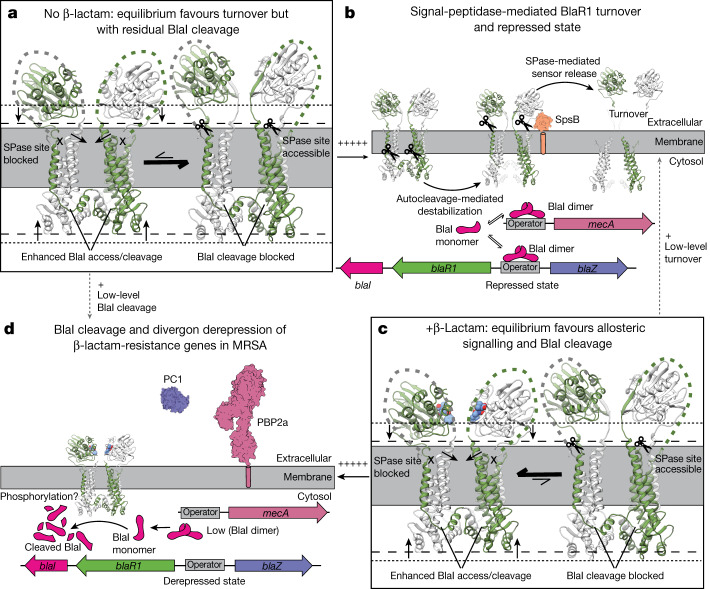
Conformational equilibrium of BlaR1 governing β-lactam resistance in MRSA. **a**, In the absence of β-lactams, BlaR1 primarily exists in the basal state with the sensor and metalloprotease domains in the most extended conformation away from the membrane outer and inner leaflets, respectively. The EL1 loop is bound within the sensor active site, the SPase cleavage site is exposed and the zinc metalloprotease domain is blocked by the autocleavage loop from efficiently cleaving BlaI. The dotted and dashed lines indicate the edge of the sensor and zinc metalloprotease domains in their extended or contracted (with β-lactam) states, respectively. **b**, In this basal BlaR1 state, elevated cytosolic BlaI concentrations favour repressor dimerization and binding to the operator sequence, minimizing the expression of genes encoding resistance proteins. In parallel, SPase cleavage of the sensor domain coupled with autocleavage-mediated destabilization of the transmembrane region promotes turnover of BlaR1 in the membrane in the absence of a β-lactam threat. **c**, Extracellular β-lactams are detected by the BlaR1 sensor through acylation of the SXXK motif, displacing EL1 and promoting movement of the sensor and the metalloprotease domains towards the membrane (Fig. 3), leading to allosteric activation. These events are thought to stabilize the active state of BlaR1 in the presence of antibiotics by blocking of SPase cleavage and promoting a more catalytically competent metalloprotease active site. Autocleavage loop proteolysis also favours BlaI cleavage, probably due to reduced steric hinderance of BlaI binding. However, autocleavage does not appear to be triggered by β-lactam, and we propose that autocleavage is instead needed for BlaR1 maturation and turnover. **d**, The activated metalloprotease domain cleaves BlaI, decreasing the concentration of BlaI dimer and reducing BlaI binding to the operator. Expression of the divergon genes *mecA* and *blaZ* leads to β-lactam protection by PBP2a (β-lactam insensitive; PDB 4WVJ shown) and PC1 (β-lactamase activity; PDB 3BLM shown), respectively.

An outstanding question of BlaR1 function is how sensing environmental β-lactams outside the cell regulates BlaI cleavage inside. The structures here describe concerted motions that link the sensor and metalloprotease domains located on opposite sides of the membrane (Fig. 3 and Supplementary Video 3). We hypothesize that the extremes are representative of changes that are involved in the activation and inhibition of the metalloprotease. Although a range of states exists in all of the samples, the equilibrium is shifted—BlaR1(WT) is dominated by a more extended conformation and the BlaR1(F284A)–ampicillin structure is more compact, with the sensor directly in contact with the outer leaflet of the membrane. In the sensor domain, the substrate-mimicking interactions of EL1 within the β-lactam-binding site and the resulting induced domain movement after displacement explain its previously predicted role in signal transduction^19,20^. On the inner leaflet, concomitant motions are observed, with the metalloprotease domain drawn up to the membrane in the presence of ampicillin. Central to this is helix α3, which traverses the membrane directly linking EL1 and the metalloprotease domain. This helix is bent into the membrane at its N terminus in the BlaR1(F284A)–ampicillin structure, seemingly as a result of the force exerted on EL1 due to the acylation-induced sensor-domain movement (Fig. 3e and Extended Data Fig. 7d). The current resolutions, in particular for the BlaR1(F284A)–ampicillin data, limit analysis of the precise structural changes. However, we note that the overall compression results in a more compact metalloprotease active site with Tyr261 (discussed above) positioned at different distances to His201, moving closer in the ampicillin-bound state of BlaR1(F284A) or in the post-cleavage state of BlaR1(WT), in which the now-free P1′ Phe284 of the cleaved product is not as deeply socketed in the S1′ subsite (Fig. 3f and Extended Data Fig. 7e). In all of the structures, the protease domain is in an autoinhibited state with the autocleavage loop (both cleaved and uncleaved) occupying the active site, suggesting an equilibrium between active site occupied/autoinhibited and active site accessible states (Fig. 4). We hypothesize that the regulation of BlaI cleavage is dependent on the equilibrium between these conformations, with both spontaneous autocleavage and β-lactam acylation serving to modulate this structural equilibrium, pushing it to one favouring displacement of the cleaved autoinhibitory loop fragments from the active site to allow efficient BlaI repressor access and cleavage after the detection of environmental β-lactam (Fig. 4).

*S. aureus* kinase (Stk1)-mediated phosphorylation of BlaR1 is involved in the activation of the *bla* pathway^51^. Tyrosine phosphorylation was specifically implicated^51^, and the only conserved cytoplasmic tyrosine residues are Tyr197, projecting from the C-terminal end of α6a, and the above mentioned Tyr261 on α8 that we observed in a spectrum of active-site-engaged positions (Fig. 3f and Extended Data Fig. 9b). Compellingly, these two side chains stack in an antiparallel manner, with Tyr197 projecting to the exterior and accessible for phosphorylation and Tyr261 towards His201. As such, it is possible that the observed conformational changes may facilitate phosphorylation, inducing added structural changes to destabilize the autocleavage loop products from the active site. Identification of phosphorylated residues and their structural effects could illuminate how phosphorylation contributes to the regulation of resistance and would lend further credence to existing efforts to develop inhibitors targeting Stk1^51^.

Another interesting aspect of BlaR1 is the need for turnover of the basal-level-expressed receptor^28^. Signal-peptidase-mediated cleavage of *Staphylococcus epidermidis* BlaR1^52^ and shedding of the sensor domain in *S. aureus* cultures at equivalent residues (between Gly331 and Gln332) have been documented^28^. We observed the presence of an analogous ~31 kDa band, which is acylated by BOCILLIN FL (Extended Data Fig. 2c). The *S. aureus* signal peptidase SpsB cleaves BlaR1(WT), with mutation of BlaR1 Gly331 to lysine abrogating this effect (Extended Data Fig. 8g). This cleavage position sits in the dynamic linker between the C-terminal transmembrane helix α10 and the sensor. In the more extended (minus β-lactam) positioning of the sensor domain away from the membrane (Fig. 3a), this loop would be more accessible for processing by membrane-anchored SpsB. Together with autocleavage, these processing events could promote destabilization of the dimeric interface of BlaR1 on both leaflets and promote inactivation/turnover to prevent potentially deleterious accumulation in the membrane and leaky BlaI cleavage/expression of the resistance genes in the absence of β-lactam. After sensing β-lactam, with reorientation of the sensor domain against the membrane (Fig. 3c), this loop is more protected from processing, maintaining BlaR1 integrity to combat the presence of the bactericidal antibiotic (Fig. 4). These observations provide further support for the ongoing interest in the development of signal peptidase inhibitors, of which the potential for synergistic effects on targets beyond the signal peptide processing roles, such as BlaR1 turnover here, is increasingly compelling.

In summary, the data presented here provide insights into the structure and function of the BlaR1 master controller of β-lactam resistance in *S. aureus*. In addition to clinically important *Staphylococcal* pathogens, analogous signalling systems have been identified in *C. difficile*^23^ and *M. tuberculosis*^22^. *C. difficile*—a notoriously persistent spore-forming pathogen—has equivalent components to the *bla* pathway (BlaR1, around 30% sequence identity with *S. aureus*; Extended Data Fig. 1) controlling the expression of a β-lactamase^23^. The *M. tuberculosis* BlaR1 homologue has a similar zinc metalloprotease domain but lacks an equivalent sensor domain making it unclear how β-lactams are detected. One possible functional substitution could involve phosphorylation by PknB, a kinase with a β-lactam-binding PASTA domain that is essential to *M. tuberculosis*^22^. We expect that these BlaR1 homologues have a similar domain-swapped architecture and proteolytic derepression of broad-spectrum resistance mechanisms, making the findings here broadly relevant in terms of mechanistic understanding and structure-guided inhibition for the preservation of an important drug class for fighting infections—the β-lactam antibiotics.

## Methods

### Plasmid construction and site-directed mutagenesis

*S. aureus* BlaR1 (UniProt: Q00419); residues 1–585) or BlaR1 from MRSA strain USA300 (RefSeq: WP_001096381.1; residues 1–585) were cloned into the *Lactococcus lactis* expression vector pNZ8048^53^ with a C-terminal hexahistidine tag, using restriction-free cloning^54^. Cloning and site-directed mutagenesis of pNZ8048 was performed in *E. coli* MC1061 grown in LB medium supplemented with 10 µg ml^−1^ chloramphenicol. After verification of the DNA sequence, plasmids were transformed into electrocompetent *L. lactis* using standard techniques^55^.

### BlaR1 protein expression

BlaR1 constructs were expressed in *L. lactis* PNZ9000 using the nisin-controlled gene expression system^25^. Expression strain seed culture was grown in Difco M17 broth (BD)^56^ supplemented with 10 µg ml^−1^ chloramphenicol and 10 g l^−1^ glucose. Large-scale protein expression was performed in a fermenter (Applicon) at 30 °C in M17 BlaR1 Expression Media (M17BEM) and inoculated with seed culture in a 1:100 (v/v) ratio. M17BEM was developed for BlaR1 protein expression based on M17 medium^56^ (Supplementary Table 2). After sterilization, the medium was supplemented with 10 µg ml^−1^ chloramphenicol and 100 µM zinc sulfate. The medium was maintained at pH 7 during fermentation with the addition of ammonium hydroxide. Cells were grown to an optical density at 600 nm of around 1.5 before nisin was added to a final concentration of 5 ng ml^−1^. Protein was expressed for 2–3 h, before collection by centrifugation, then the cells were frozen in liquid nitrogen and stored at −80 °C.

### BlaR1 purification

Frozen cell pellets were resuspended in lysis buffer (150 mM sodium chloride; 50 mM HEPES, pH 7.5) with 5 mg ml^−1^ lysozyme (Bio Basic) and 20 µg ml^−1^ DNase I (Roche) and incubated at 37 °C for 1 h with stirring. The cells were then cooled in an ice bath before being lysed with a single pass using a French press (Thermo Fisher Scientific) at ~25,000 psi. All of the subsequent steps were carried out at 4 °C. The lysate was centrifuged at 18,600*g* for 30 min. The supernatant was collected and centrifuged at 40,000 rpm in a Type 45 Ti rotor (Beckman Coulter) for 1 h to pellet the membranes. The membranes were resuspended in lysis buffer using a glass Teflon Dounce homogenizer before being frozen in liquid nitrogen and stored at −80 °C until needed.

Membranes were thawed and resuspended in lysis buffer with 1% (w/v) *n*-dodecyl-β-d-maltopyranoside (DDM) for 1 h to extract the membranes. All detergents used here were acquired from Anatrace. The extracted membranes were centrifuged at 40,000 rpm in a Type 45 Ti rotor (Beckman Coulter) for 45 min to pellet insolubilized membranes. The supernatant was then filtered through a 0.45 µm membrane and imidazole at pH 7.5 was added to 20 mM before being loaded onto a 1 ml HisTrap HP (GE healthcare) equilibrated in buffer A (150 mM sodium chloride; 20 mM HEPES, pH 7.5; 0.016% (w/v) Anagrade DDM or 0.01% (w/v) lauryl maltose neopentyl glycol (LMNG)) supplemented with 20 mM imidazole at 0.5 ml min^−1^. The column was then sequentially washed with 15 ml buffer A with 20 mM imidazole, 15 ml buffer A with 100 mM imidazole, and eluted in buffer A with 325 mM imidazole. The protein was then immediately desalted into buffer A with a XK-26 column (Amersham Biosciences) packed with Sephadex G-25 medium resin (GE healthcare). Protein was then concentrated using a stirred-cell concentrator with a 100 kDa molecular mass cut-off cellulose membrane (Merck Millipore) before continued purification or flash-freezing in liquid nitrogen followed by storage at −80 °C. BlaR1 concentration was determined by absorbance at 280 nm using the theoretical extinction coefficient, 106,245 M^−1^ cm^−1^, calculated using ExPASy^57^.

During purification of BlaR1, a band directly below the main full-length band was observed on the SDS–PAGE gel (Extended Data Fig. 2e). Edman N-terminal sequencing revealed cleavage was occurring between BlaR1 residues Ser72 and Lys73, corresponding to an exposed region of EL1 and loss of transmembrane helices 1 and 2. As this minor cleavage product has not to our knowledge been observed in BlaR1 expressed in *S. aureus* we expect that this cleavage is due to proteases originating in the *L. lactis* expression system used. We did not see any evidence of this cleavage product in our cryo-EM subclasses or reconstructions.

For cryo-EM, BlaR1 samples in LMNG were further purified using the GraDeR^58^ rate zonal ultracentrifugation method. Specifically, 5–25% (v/v) glycerol gradients (light buffer: 5% (v/v) glycerol, 150 mM sodium chloride, 20 mM HEPES, pH 7.5 with or without 0.01% LMNG detergent; heavy buffer: 25% (v/v) glycerol, 150 mM sodium chloride, 20 mM HEPES, pH 7.5) were made using a Gradient Master (BioComp Instruments)^59^. For samples with ampicillin, BlaR1(F284A) was incubated for around 15 min on ice with 1 mM ampicillin and then all subsequent buffers were made with 250 µM ampicillin. After overlaying 150–250 µl of protein sample onto the glycerol gradients, they were immediately centrifuged for the equivalent of 8 h at 55,000 rpm in a SW 55 Ti rotor (Beckman Coulter) at 4 °C. The gradients were then fractionated using a gradient fractionator (BioComp Instruments). The relevant fractions were concentrated using an Amicon Ultra 100 kDa molecular mass cut-off centrifugal filter (Merck Millipore) at 6,000*g* and 4 °C, before being desalted using a Micro Bio-Spin P-30 Chromatography Column (Bio-Rad Laboratories) into buffer B (150 mM sodium chloride and 20 mM HEPES, pH 7.5). The purity was checked on SDS–PAGE gels. For some gels, BlaR1 was incubated with a final concentration of 50 µM BOCILLIN FL^27^ for approximately 5 min at room temperature before the addition of loading dye to fluorescently label the sensor domain.

### Edman N-terminal sequencing

Samples for Edman N-terminal sequencing were separated on an SDS–PAGE gel before being transferred to a polyvinylidene fluoride membrane and stained using Coomassie. Edman N-terminal sequencing was performed at the SPARK BioCentre.

### BlaR1 activity assay with BlaI substrate

#### Purification of T7-tagged BlaI

The full-length *blaI* gene (UniProt: P0A042) from *S. aureus* was cloned into pET21a with an N-terminal T7-tag using restriction-free cloning^54^. This plasmid was transformed into electrocompetent *E. coli* Rosetta (DE3) cells. Transformed cells were grown at 37 °C in LB medium supplemented with 100 µg ml^−1^ ampicillin. At an OD_600_ of around 0.6–0.8 protein expression was induced by the addition of isopropyl β-d-1-thiogalactopyranoside (IPTG) to 1 mM and the temperature was lowered to 20 °C. Cells were allowed to express protein overnight before collection, flash-freezing in liquid nitrogen and storage at −80 °C.

Protein purification was performed at 4 °C with adaptations from a previous protocol^60^. Cells were resuspended in buffer 2A (50 mM HEPES, pH 7.0) with 1 cOmplete EDTA-free protease inhibitor cocktail tablet (Roche) and DNase I to ~20 µg ml^−1^ and lysed in a French press at 16,000–25,000 psi. BlaI was then purified by ammonium sulfate precipitation with sequential addition of ammonium sulfate to 50%, 60%, 70% and 80% saturation. After each addition of ammonium sulfate, the lysate was incubated for 15 min with inversion followed by centrifugation to pellet the precipitated protein. Protein pellets from 70% and 80% saturated ammonium sulfate solutions were resuspended in buffer 2A and dialysed overnight in buffer 2B (50 mM HEPES, pH 7.0; 5% glycerol). The protein was then loaded on a 5 ml Heparin column (GE Healthcare) equilibrated in buffer 2B at 1 ml min^−1^. The column was washed with buffer 2B until the absorbance at 280 nm stabilized. Protein was eluted from the column with a linear gradient of buffer 2B to buffer 2C (50 mM HEPES, pH 7.0; 5% glycerol; 2 M sodium chloride) over 60 min at a flow rate of 2 ml min^−1^. Fractions of interest were concentrated on an Amicon Ultra 10 kDa molecular mass cut-off centrifugal filter (Merck Millipore) and loaded onto a Superdex 75 10/300 column (GE healthcare) equilibrated in buffer 2D (20 mM HEPES, pH 7.0; 500 mM sodium chloride; 5% glycerol). Purified protein was concentrated as before to 4 mg ml^−1^ (as determined by absorbance at 280 nm and correction using the theoretical extinction coefficient 19,940 M^−1^ cm^−1^ calculated using the ExPASy server)^57^ before being frozen in liquid nitrogen and stored at −80 °C until needed.

#### Assaying BlaR1 activity with purified, T7-tagged BlaI

BlaR1(WT), BlaR1(F284A) or BlaR1(USA300) was incubated with BlaI for 4 h at room temperature in assay buffer (150 mM sodium chloride, 20 mM HEPES, pH 7.5, 0.016% DDM). After the incubation period, the reaction was quenched with SDS loading buffer and the samples were analysed using western blotting. The western blot was probed with an anti-T7-tag antibody HRP conjugate (Novagen) diluted 1:15,000 in TTBS (20 mM Tris, 150 mM NaCl, 0.1 % Tween-20) and 3% non-fat skimmed milk powder) for 1 h at room temperature. The blot was developed using Western Chemiluminescent HRP Substrate (Immobilon) and imaged using the Chemi Genius 2 Bio Imaging System (Syngene).

#### Determining the BlaI cleavage site

To aid Edman N-terminal sequencing, a BlaI–maltose-binding protein (MBP) fusion construct was cloned to the C terminus of BlaI with a Gly-Ser-Ser linker. Initial attempts to transfer the 3 kDa fragment onto the polyvinylidene fluoride membrane failed, but when fused to MBP, the transfer was trivial. The BlaI-MBP construct was designed with an N-terminal decahistidine tag and was cloned into a pGEX-6P-1 expression plasmid with restriction-free cloning^54^.

Expression was performed as described for the T7-tagged BlaI construct and cells were lysed as before in buffer 3A (25 mM Tris, pH 8, and 100 mM sodium chloride) with 1 cOmplete EDTA-free protease inhibitor cocktail tablet (Roche) and DNase I (Roche) to ~20 µg ml^−1^. The cell lysate was centrifuged at 40,000 rpm in a type 45 Ti rotor (Beckman Coulter) for 45 min at 4 °C. The supernatant was filtered through a 0.45 µm filter and imidazole was added to 10 mM before the sample was loaded onto a 1 ml HisTrap HP (GE Healthcare) equilibrated in buffer 3A at 0.75 ml min^−1^. The column was then washed with 10 cv of 2% buffer 3B (50 mM Tris pH 8, 500 mM imidazole and 100 mM sodium chloride), before being eluted on a gradient of 0–100% over 40 min at flow rate of 1 ml min^−1^. Fractions containing the protein of interest were concentrated on an Amicon Ultra 30 kDa molecular mass cut-off centrifugal filter (Merck Millipore). The concentrated protein was loaded onto the Superdex 75 10/300 column (GE healthcare) equilibrated in buffer 3C (25 mM Tris, pH 8.0, 100 mM sodium chloride). Purified protein was concentrated as before, frozen in liquid nitrogen and stored at −80 °C until needed.

### Inductively coupled plasma mass spectrometry

BlaR1(F284A) was purified in LMNG using immobilized metal affinity chromatography before being concentrated and purified by SEC in buffer B. Protein and buffer samples were prepared in 1% HNO_3_ with ^45^Sc (100 ppb) as an internal standard. Inductively coupled plasma mass spectrometry to determine zinc concentration was performed using the NexION 300D (Perkin Elmer) system equipped with a SC-2 DX autosampler, DXi-FAST micro-peristaltic pump, a cyclonic spray chamber, a triple cone interface, a quadrupole ion deflector and Universal Cell Technology. Calibration was performed using IV-Stock-4 calibration standard (Inorganic Ventures). All elements were run in reaction mode (using Dynamic Reaction Cell technology) using ammonia as a reaction gas to remove potential polyatomic interferences. The detection limit for ^66^Zn was determined to be 0.765 ppb.

### Cryo-electron microscopy of BlaR1

For all cryo-EM datasets, C-flat (1.2/1.3) 300 mesh grids (Protochips) were glow-discharged before 3 µl of protein sample at an absorbance at 280 nm of 0.27–0.35 was applied to the grid. The grids were blotted for 1–2 s using the Vitrobot Mark IV (Thermo Fisher Scientific) at 4 °C and 100% humidity before being plunged into liquid ethane. The grids were transferred to liquid nitrogen for storage until imaging.

Cryo-EM grids were screened at the High-resolution Macromolecular Cryo-Electron Microscopy facility at the University of British Columbia on a Glacios (Thermo Fisher Scientific) transmission cryogenic electron microscope with a 200 kV accelerating voltage and a Falcon 3 camera using the EPU (Thermo Fisher Scientific) software package.

The BlaR1(WT) dataset was collected at the High-resolution Macromolecular Cryo-Electron Microscopy facility at the University of British Columbia using the 300 kV Titan Krios (Thermo Fisher Scientific) transmission cryogenic electron microscope equipped with a Falcon 4i (Thermo Fisher Scientific) direct electron detector and a Selectris (Thermo Fisher Scientific) energy filter. Images were gathered in electron-counting mode with a calibrated magnification of ×165,000, corresponding to 0.77 Å per physical pixel. The total dose delivered to the grids was 50 e^−^ Å^−2^ over 875 frames in the EER video format. Fully automated data collection was performed using EPU (Thermo Fisher Scientific) with a nominal defocus range set from 0.5 to 3.0 μm.

The BlaR1(F284A) ligand-free dataset was collected at the Pacific Northwest Cryo-EM Centre using a 300 kV Titan Krios (Thermo Fisher Scientific) transmission cryogenic electron microscope equipped with the GIF Quantum energy filter (Gatan; operated at 20 keV) and a K3 Summit (Gatan) direct electron detector. Images were gathered in super-resolution mode at a calibrated magnification of 81,000x, corresponding to 1.059 Å per physical pixel (0.5295 Å per super-resolution pixel). A total dose of 33 e^−^ Å^−2^ was delivered to the grids over 51 frames. Fully automated data collection was performed using SerialEM with a nominal defocus range set from 1.5 to 3.5 μm (ref. ^61^).

The BlaR1(F284A)–ampicillin dataset was collected at the HHMI Janelia Research Campus using the 300 kV Titan Krios (Thermo Fisher Scientific) transmission cryogenic electron microscope equipped with a spherical aberration corrector, a GIF Quantum energy filter (Gatan) operated at 20 keV and a K3 Summit (Gatan) direct electron detector. Images were gathered in super-resolution mode at a calibrated magnification of ×64,000, corresponding to 1.065 Å per physical pixel (0.5325 Å per super-resolution pixel). The total dose delivered to the grids was 60 e^−^ Å^−2^ over 60 frames. Fully automated data collection was performed using SerialEM with a nominal defocus range set from 1.0 to 2.0 μm (ref. ^61^).

### Cryo-EM data processing

For the BlaR1(WT) sample, 16,599 videos were collected at a pixel size of 0.77 Å per pixel. Motion correction was performed using MotionCor2^62^ and the contrast transfer functions (CTFs) of the summed and dose-weighted micrographs were determined using CTFFIND4^63^. Approximately 2,000 particles were manually selected to generate reference-free 2D-class averages in RELION^64^. Representative 2D classes were used as templates for automated particle picking in RELION (v.3.1 and v.4) with 7,508,828 particles picked. 2D classification was performed using CryoSPARC (v.3.2 and v.3.3.2)^65^, resulting in 4,286,427 particles taken forward for further processing. Three initial volumes were generated and processed for multiple rounds of heterogeneous refinement. The best volume was further refined with NU-refinement with *C*_2_ symmetry imposed and used as the initial model for additional rounds of heterogenous refinement to recover good particles that were previously discarded in the 2D classification steps. A particle dataset of 286,692 particles was transferred back to RELION with cs2star in the pyem^66^ software package and processed for multiple rounds of 3D classification. The particles associated with the best volumes were grouped, refined and used for Bayesian polishing^67^ and CTF refinement^68^. Partial signal subtraction was used to remove the micelle density. A reconstruction with *C*_2_ symmetry applied was generated from 143,227 particles to a resolution of 4.2 Å as determined using the gold-standard refinement procedure with an FSC of 0.143 and high-resolution noise substitution to correct soft-mask effects^69^. Symmetry expansion followed by further 3D classification without alignment using a mask covering the sensor domains identified different sensor domain states. A representative class with the most resolved EL1 region contained 23,797 unique particles and refined to 4.9 Å resolution with no symmetry applied (*C*_1_). Signal subtraction to remove the sensor domain density was carried out on the 143,227 particles again followed by symmetry expansion and 3D classification without alignment resulting in 66,347 unique particles that refined to 3.8 Å resolution with no symmetry applied (*C*_1_) (Extended Data Table 1 and Extended Data Figs. 3 and 4).

For the BlaR1(F284A) sample, a total of 11,844 video series was collected in super-resolution mode at 0.5295 Å per pixel and binned to 1.059 Å per pixel during motion correction performed with MotionCor2^62^. The CTFs of the summed and dose-weighted micrographs were determined using CTFFIND4^63^. Particle picking was carried out as described above with 4,181,855 particles automatically picked. Reference-free 2D classification was performed in CryoSPARC^65^, resulting in 534,610 good particles taken for further processing. Further particle sorting was performed as described above and 221,007 particles were transferred back to RELION and processed for multiple rounds of 3D classification, and the particles associated with the best volumes were grouped, refined, and used for Bayesian polishing^67^ and CTF refinement^68^, and the micelle density was removed through partial signal subtraction. One round of 3D classification without alignment was performed and the particles belonging to the best volumes were grouped and refined. The resulting volume after postprocessing reached a resolution of 4.3 Å with *C*_2_ symmetry averaging applied (Extended Data Fig. 3). An example data-processing overview is included in Extended Data Fig. 5.

For the BlaR1(F284A)–ampicillin sample, 10,872 video series were collected in super-resolution mode at 0.5325 Å per pixel and binned to 1.065 Å per pixel during motion correction performed using MotionCor2^62^. The CTFs of the summed and dose-weighted micrographs were determined using CTFFIND4^63^. Particle picking was performed as described above with 5,613,933 particles automatically picked. Reference-free 2D classification was performed in CryoSPARC^65^, resulting in 216,832 good particles. Further particle sorting was carried out using heterogeneous refinement as described above and 245,700 particles were transferred back to RELION. Multiple rounds of 3D classification were carried out with the particles associated with the best volumes grouped, refined, used for Bayesian polishing^67^ and CTF refinement^68^, and the micelle density was removed through partial signal subtraction. One round of 3D classification without alignment was performed and the particles belonging to the best volumes were grouped and refined. The resulting volume after post-processing reached a resolution of 4.6 Å with *C*_2_ symmetry averaging applied (Extended Data Fig. 3).

### Model building and refinement

A prediction of the transmembrane and catalytic regions of the BlaR1 structure from HHPred^70^ and Modeller^71^ was used as a starting point for model building using the BlaR1(WT) transmembrane reconstruction to 3.8 Å resolution. Incorrect regions were deleted and iterative model building and refinement carried out in Coot^72^, Phenix real.space refine^73^ in the Phenix software package^74^, and density-guided Rosetta refinement with symmetry^39^. For the sensor domain, the X-ray crystal structure of the *S. aureus* sensor domain (PDB: 1XA7) was automatically fit into the 4.2 Å *C*_2_ reconstruction using Phenix dock_in_map. Although this region was of lower local resolution (Extended Data Fig. 3), clear secondary structure features enabled confident placement (Extended Data Fig. 7a–c). Residues 52–96, forming the extracellular facing loop EL1, were not clearly resolved in entirety. AlphaFold2 (not available at the time of initial model building) reproducibly and confidently predicted the C-terminal region of this loop as bound to the sensor-domain β-lactam binding groove, which was supported by low-resolution features in the reconstruction (Extended Data Fig. 6g–i). The EL1 loop conformation for residues 87–97 from AlphaFold2 was incorporated into the final model and subject to extensive density restrained refinement in Rosetta and Phenix real.space refine. Sensor-domain loop 413–427 was omitted due to interchain clashes. For the BlaR1(WT) *C*_1_ (4.9 Å) reconstruction with shifted sensor-domain positions, residues 413–427 did not clash and were included. For the BlaR1(F284A) (4.3 Å) and BlaR1(F284A)–ampicillin (4.6 Å) reconstructions, the transmembrane/catalytic regions and sensor domains were docked into the density. The relative distance between sensor and transmembrane domains was different between BlaR1(WT) (furthest), BlaR1(F284A) and BlaR1(F284A)–ampicillin (closest) with the loop (residues 332–345) joining the two adopting different relative positions. For BlaR1(F284A), the final model consists of residues 1–55 and 87–585 and, for BlaR1(F284A)–ampicillin, residues 1–55, 97–412 and 428–585 (EL1 residues 87–96 and sensor-domain residues 413–427 were omitted). Two active site zincs were included in both cases (Extended Data Table 1 and Extended Data Figs. 3, 4 and 6).

Model validation was performed using MolProbity as implemented in Phenix^75,76^. Figures were created using PyMol (Schrödinger), UCSF Chimera^77^, UCSF ChimeraX^78^ and Adobe Illustrator 2021 (Adobe). BlaR1 was inserted into a membrane generated with the CHARM-GUI membrane builder^79^. Protein interface analysis was carried out with the PISA server using the default parameters^80^. Sequence conservation was analysed using the ConSurf server^81^.

### BlaR1 functional assays carried out in *S. aureus*

The *S. aureus* strains used in the assay (Supplementary Table 3) were cultured in tryptic soy broth (TSB) at 37 °C at 180 rpm. The natural plasmid of SF8300 (a USA300 strain) that contains the *blaR1-blaI* regulatory genes (pUSA300HOUMR) was used as a template for PCR amplification. Different variants of the *blaR1-blaI* constructs were generated by splice overlap PCR and cloned into a constitutively expressing vector, pTX_Δ_^82^. The plasmids were then transformed to RN4220 through electroporation. After sequence verification, the plasmids were introduced to SF8300ermS (erythromycin sensitive SF8300) through transduction using the phage, Φ11. SF8300ermS was obtained by evicting pUSA300HOUMR through serial passaging of SF8300 in TSB and by selecting for an erythromycin-sensitive colony^83^. pUSA300HOUMR, in addition to containing *blaZ* and its control elements (*blaR1-blaI*), also contains an erythromycin-resistance gene^84^. Thus, the SF8300ermS lacked β-lactamase and its control elements (*blaZ-blaR1-blaI*). Lists of the plasmids and primers used in this assay are provided in Supplementary Tables 4 and 5, respectively. Strains containing the pTX_Δ_ plasmid were grown and maintained in 12.5 µg ml^−1^ tetracycline. *blaR1* and *blaI* are expressed from a plasmid and in turn control the expression of chromosomally encoded *mecA*. For this purpose, *blaR1(F284A)-blaI* was cloned into a constitutively expressed plasmid and transduced into SF8300ermS that lacked the natural plasmid (pUSA300HOUMR) containing *blaZ* and its regulatory elements (*blaR1-blaI)*. SF8300ermS transduced with an identical plasmid with either an empty vector, a fully functional *blaR1-blaI* or a functionally attenuated *blaR1(null)-blaI* and *blaR1(null)-blaI(null)* constructs were used as controls. The null mutants were created by mutating the methionine start codon of *blaR1* and *blaI* to threonine, leading to strains deficient of these proteins. The isogenic strains thus created were assessed for their growth properties either in absence or in presence of nafcillin, an easily available and prototype β-lactam antibiotic that is structurally similar to methicillin.

Growth assays were performed using the Bioscreen C Pro system (Growth Curves USA). The initial bacterial OD_600_ was adjusted to 0.1 in TSB containing 2 µg ml^−1^ nafcillin or no nafcillin and 200 µl of this mixture was added to a Honeycomb microplate (Growth Curves USA) in triplicates. Wells containing TSB without any antibiotics were included as controls. The readings were recorded every 30 min for 24 h at 600 nm. The temperature was set to 37 °C. The settling time was set to 30 s and the shake duration was 15 s. DNA sequence analysis was performed using DNAstar software. All experiments except for sequencing were repeated at least twice. The fidelity of all the plasmid constructs and point mutations described in this study were validated through Sanger sequencing.

### BlaR1 cleavage with *S. aureus* signal peptidase SpsB

The gene for *S. aureus* MRSA252 SpsB (UniProt: Q6GIC3) was cloned into the expression vector pET28a+ (Novagen) using the NdeI and XhoI sites. The expressed protein has an N-terminal hexahistidine tag, followed by a thrombin cleavage site (MGSSHHHHHHSSGLVPRGSHM). Plasmid was transformed into *E. coli* BL21(DE3). One litre of LB medium supplemented with kanamycin (0.05 mg ml^−1^) was inoculated with 50 ml of overnight culture. Cells were grown at 37 °C with shaking (250 rpm) until an OD_600_ of 0.6, at which point 1 mM of IPTG was added to the culture to induce protein expression. Cells were grown for 4 h after induction, then pelleted by centrifugation (6,000*g*, for 5 min at 4 °C). Then, 5 g of the cell pellet was resuspended in 25 ml of buffer A (20 mM Tris pH 8.0, 100 mM NaCl) and then sonicated using a Sonic Dismembrator Model 500 (Thermo Fisher Scientific), with a 15 s pulse and 30 s rest cycle three times at 30% amplitude, on ice and lysed by passing through an EmulsiFlex-C3 cell disruptor (Avestin) for 5 min. The resulting cell lysate was clarified by centrifugation (35,000*g* for 30 min at 4 °C). The pellet was resuspended in 25 ml of buffer A with 0.1% Triton X-100 and dounced 50 times followed by centrifugation (35,000*g* for 30 min at 4 °C). The clarified supernatant was then subjected to Ni^2^^+^-NTA affinity chromatography purification. The clarified supernatant, containing the His-tagged SpsB, was poured onto a column containing 3 ml of Ni^2+^-charged resin (Qiagen) pre-equilibrated with kinetic buffer (buffer A with 0.01% DDM). The column was washed twice with 30 ml of kinetic buffer containing first 10 mM, and then 20 mM imidazole. Next, His-tagged SpsB was eluted using a step gradient, created by 6 ml of kinetic buffer containing imidazole in increasing concentrations of 100 to 500 mM imidazole. Fractions containing purified SpsB were concentrated to 1 ml using an Amicon ultracentrifugal filter (Millipore) with a 3 kDa cut-off and dialysed against 1 l of kinetic buffer at 4 °C overnight. Protein concentration was determined by BCA assay (Thermo Fisher Scientific) according to the manufacturer’s protocol.

To assay for SpsB-mediated BlaR1 cleavage, BlaR1(WT) or BlaR1(G331K) in reaction buffer (150 mM sodium chloride; 20 mM HEPES, pH 7.5, 0.01% (w/v) LMNG) was incubated overnight at room temperature with an excess molar ratio of SpsB. The resulting sample was incubated with BOCILLIN-FL^27^ for 5 min before SDS–PAGE.

### Reporting summary

Further information on research design is available in the Nature Portfolio Reporting Summary linked to this article.

## Online content

Any methods, additional references, Nature Portfolio reporting summaries, source data, extended data, supplementary information, acknowledgements, peer review information; details of author contributions and competing interests; and statements of data and code availability are available at 10.1038/s41586-022-05583-3.

### Supplementary information

Supplementary Information Supplementary Fig. 1 and Supplementary Tables 1–5.

Reporting Summary

Supplementary Video 1 Overview of BlaR1 dimer structure. One protomer is shown in green ribbon and the other in surface coloured according to electrostatics (Fig. 1d). Residues important to signalling are shown as ball and stick. Positively charged residues close to the membrane are shown as sticks. 00:00–00:14, 360 degree rotation. 00:18–00:34, close up of the active site. The autocleavage loop is coloured in darker green. 00:40–01:00, sensor-binding site showing EL1 bound in the sensor domain β-lactam-binding groove.

Supplementary Video 2 Structural variation in the BlaR1(WT) sensor domain and zinc metalloprotease active site. Morph between different sensor domain states observed for BlaR(WT). The low-pass filtered BlaR1(WT) *C*_1_reconstruction is shown in grey (solid and transparent), with the model in ribbon with one protomer coloured green and the other coloured grey. 00:11–00:25, close up morph between the two zinc metalloprotease active site states observed in the 3.8 Å BlaR1(WT) reconstruction representing pre-cleavage, with near continuous density along autocleavage loop, and post-cleavage, with clear interrupted density. The distance between Tyr261 and His231 is indicated by a blue dashed line.

Supplementary Video 3 β-lactam-induced conformational changes in BlaR1. Front view morph between BlaR1(F284A) structures without (orange) and with (cyan) ampicillin bound in the sensor domain. Maps are coloured according to Fig. 3 and are shown as solid and transparent surfaces with the structural model shown in ribbon and the protomers coloured green and grey. 00:11–00:15, side view. 00:18–00:22, slabbed (mid-point) side view. 00:25–00:31, animation of β-lactam binding and induced sensor domain movement.

## Figures and Tables

**Extended Data Table 1:**
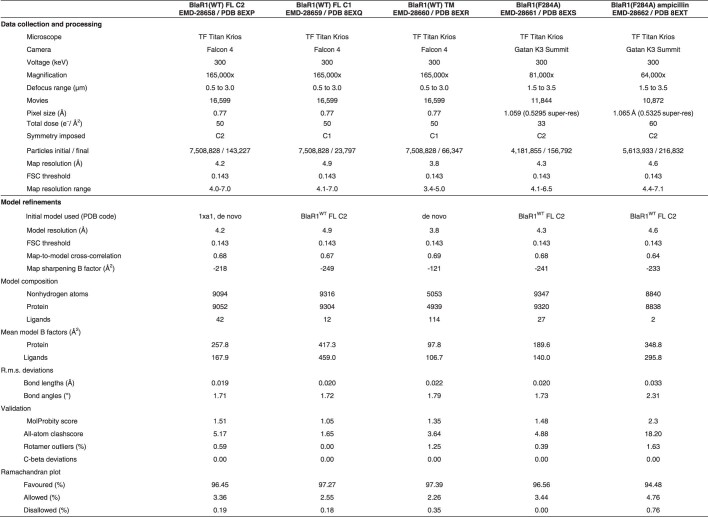
*S. aureus* BlaR1 data collection and processing statistics

## Data Availability

Models and cryo-EM reconstructions for BlaR1(WT), BlaR1(F284A) ampicillin-free and BlaR1(F284A) ampicillin-bound samples have been deposited at the PDB and the EMDB under accession codes 8EXP, 8EXQ, 8EXR, 8EXS and 8EXT, and EMD-28658, EMD-28659, EMD-28660, EMD-28661 and EMD-28662, respectively.
