# Supplementary material for: Structural basis of broad-spectrum β-lactam resistance in Staphylococcus aureus
Source: Nature. 2023 Jan 4;613(7943):375–82. doi: 10.1038/s41586-022-05583-3 (PMC9834060; doi:10.1038/s41586-022-05583-3)
Supplement: Supplementary file 1 — Supplementary Fig. 1 and Supplementary Tables 1–5. [file 41586_2022_5583_MOESM1_ESM.pdf]

nature portfolio

https://doi.org/10.1038/s41586-022-05583-3

Supplementary information

Structural basis of broad-spectrum β-lactam resistance in *Staphylococcus aureus*

In the format provided by the authors and unedited

Supplementary Information

Structural basis of broad spectrum β-lactam resistance in *Staphylococcus aureus*

J. Andrew N. Alexander[#1], Liam J. Worrall[#1,2], Jinhong Hu[1], Marija Vuckovic[1], Nidhi Satishkumar[3], Raymond Poon[3], Solmaz Sobhanifar[1], Federico I. Rosell[1], Joshua Jenkins[1], Daniel Chiang[5], Wesley A. Mosimann[1], Henry F. Chambers[4], Mark Paetzel[5], Som S. Chatterjee[3], Natalie C.J. Strynadka*[1,2]

[1]Department of Biochemistry and Molecular Biology and Centre for Blood Research, The University of British Columbia, Vancouver, British Columbia, Canada V6T 1Z3; [2]HRMEM Facility, The University of British Columbia, Vancouver, British Columbia, Canada V6T 1Z3; [3]Department of Microbial Pathogenesis, School of Dentistry, University of Maryland, Baltimore, MD and Institute of Marine and Environmental Technology, Baltimore, MD, USA 21202; [4]Division of Infectious Diseases, School of Medicine, University of California San Francisco, San Francisco, CA, USA 94110; [5]Department of Molecular Biology and Biochemistry, Simon Fraser University, Burnaby, British Columbia, Canada V5A 1S6.

[#]These authors contributed equally to this work

*Correspondence should be addressed: Natalie C.J. Strynadka: Department of Biochemistry and Molecular Biology, The University of British Columbia, Life Sciences Centre, 2350 Health Sciences Mall, Vancouver, BC V6T 1Z3, Canada. ncjs@mail.ubc.ca; Tel. +1 (604) 822-7729; Fax: +1 (604) 822-5227.

Keywords: Antibiotic resistance, β-lactam antibiotics, methicillin-resistant *Staphylococcus aureus* (MRSA), BlaR1, MecR1, cryo-EM, transmembrane signalling

Supplementary Information contains:

- Supplementary Figure 1
- Supplementary Tables 1-5

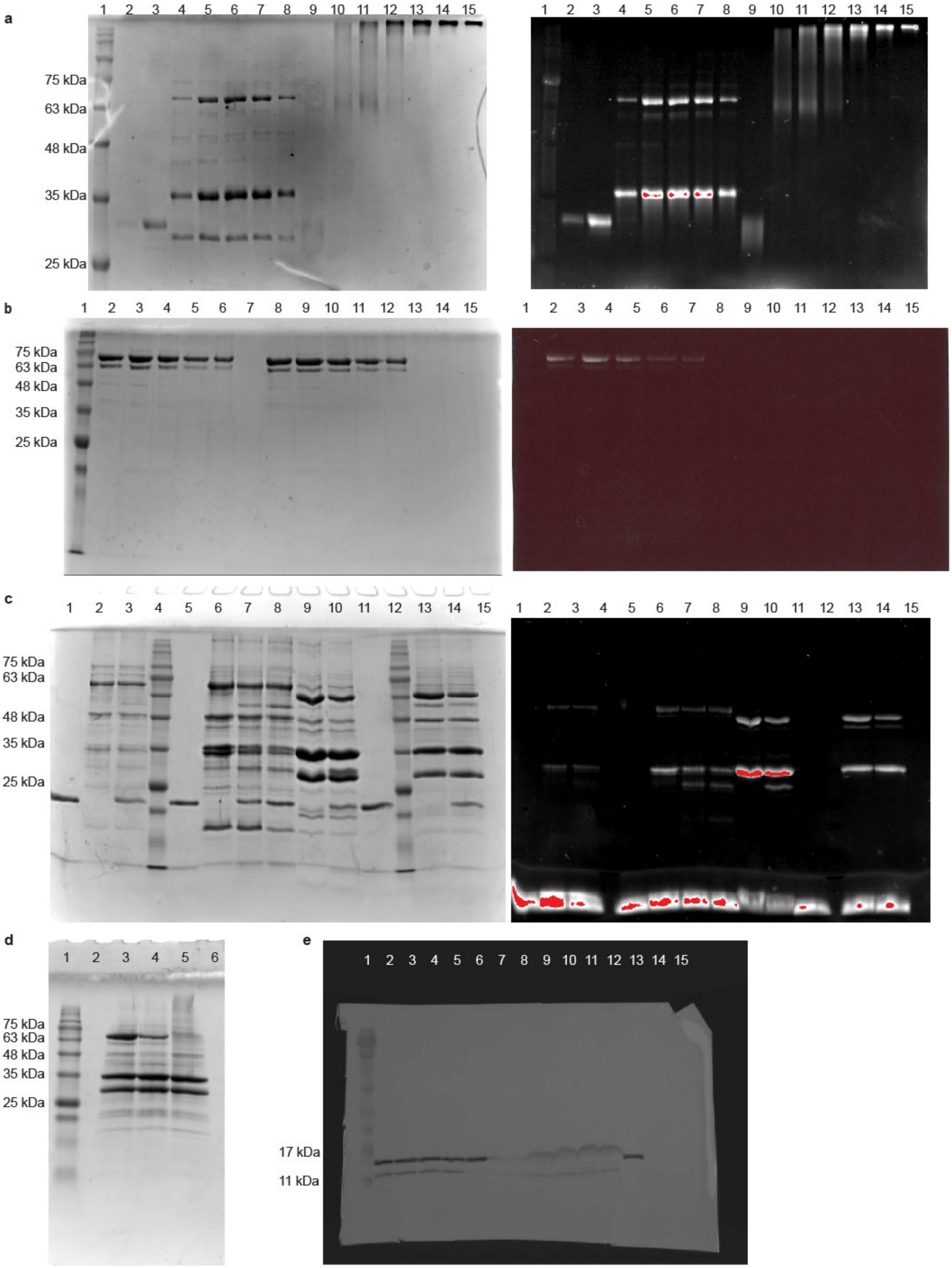
a
1 2 3 4 5 6 7 8 9 10 11 12 13 14 15
75 kDa
63 kDa
48 kDa
35 kDa
25 kDa
b
75 kDa
63 kDa
48 kDa
35 kDa
25 kDa
c
75 kDa
63 kDa
48 kDa
35 kDa
25 kDa
d
1 2 3 4 5 6
75 kDa
63 kDa
48 kDa
35 kDa
25 kDa
e
17 kDa
11 kDa

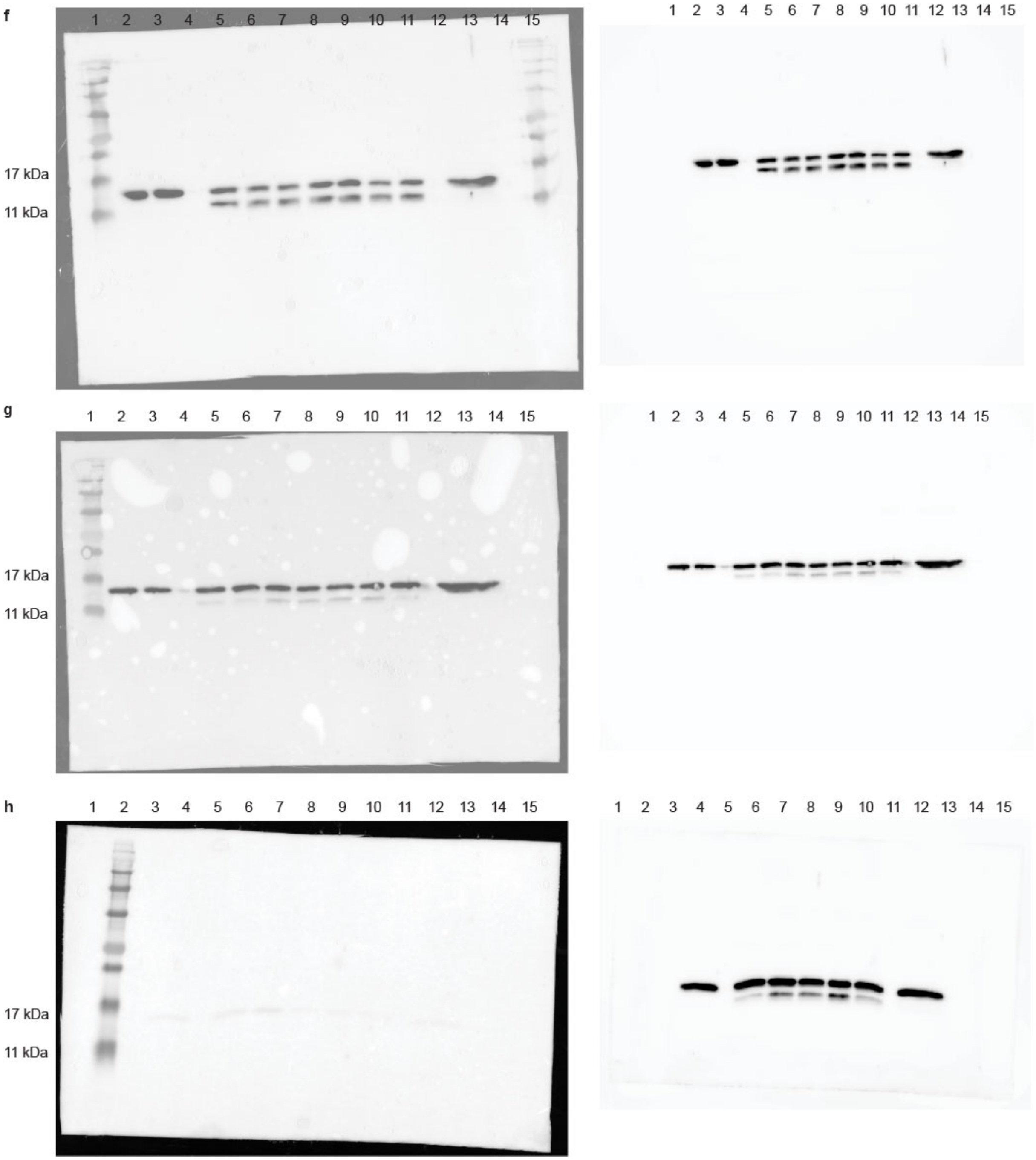

Supplementary Figure 1: Uncropped gels and blots. a, Coomassie stained (left) and fluorescent imaged (right) SDS-PAGE gels shown in Extended Data Fig. 1c. Figure cropped to show lanes 1-8. Marker is in lane 1. **b**, Coomassie stained (left) and fluorescent imaged (right) SDS-PAGE gels shown in Extended Data Fig. 1e. Figure shows full gels. Marker is in lane 1. **c**, Coomassie stained (left) and fluorescent imaged (right) SDS-PAGE gels shown in Extended Data Fig. 8g. Figure cropped to show lanes 4 (marker), 5, 9,10, 13, 14. **d**, Coomassie stained SDS-PAGE gel shown in in Extended Data Fig. 1g. Figure shows cropped lanes 1-6. **e**, Fluorescent imaged Western blot membrane shown in Extended Data Fig. 1a. Figure shows cropped lanes 1 (marker), 9, 13, 14. **f**, Colorimetric (left) and fluorescent (right) imaged Western blot membrane shown in Extended Data Fig. 8d. Fluorescent image cropped to show region around 11-17 kDa for lanes 3-14. **g**, Colorimetric (left) and fluorescent (right) imaged Western blot membrane shown in Extended Data Fig. 8e. Fluorescent image cropped to show region around 11-17 kDa for lanes 3-14. **h**, Colorimetric (left) and fluorescent (right) imaged Western blot membrane shown in Extended Data Fig. 8d. Fluorescent image cropped from to show region around 11-17 kDa for lanes 4-13.

Supplementary Table 1: ICP-MS measurement of zinc in BlaR1

Sample	[BlaR1-F284] (nM)	[Zn] (ppb)	[Zn] (nM)	Zn : Protein Ratio (mol:mol)
Measured BlaR1-F284A (Concentration corrected from buffer)	700	52 (50)	790 (760)	1.1
Buffer	-	2	30	-

Supplementary Table 2: M17BEM components prior to autoclaving

Component	Mass per litre (g)	Supplier
Glucose	10	Sigma, G8270
Bio-Tryptone	5	BioShop, TRP402
Bacto Soytone	5	BD, 243620
BBL Beef extract powder	5	BD, 212303
Yeast extract	2.5	BioShop, YEX401.205
Ascorbic acid	0.5	Bio Basic, AB0021
$MgSO_4$ (anhydrous)	0.25	Fisher, M65-500

Supplementary Table 3: List of strains used for *S. aureus* growth assay.

Strain	Description	Reference
RN4220	Laboratory strain	BEI Resources
SF8300ermS	SF8300 strain cured of the plasmid containing BlaZ-*blaR1*-*blaI* and EM resistance	[2]
RN4220 *pTxΔ*16	RN4220 with the *pTX*$_{\Delta}$16 empty vector	
RN4220 *pTX*$_{\Delta}$ [*blaR1*-*blaI*]	RN4220 with *pTX*$_{\Delta}$ containing a functional *blaR1* and *blaI*	This Study
RN4220 *pTX*$_{\Delta}$ [*blaR1*(null)-*blaI*]	RN4220 with *pTX*$_{\Delta}$ containing a functional *blaI* and a *blaR1* null mutant.	This Study
RN4220 *pTX*$_{\Delta}$ [*blaR1*(null)-*blaI*(null)]	RN4220 with *pTX*$_{\Delta}$ that contains null mutants of *blaR1* and *blaI*.	This Study
RN4220 *pTX*$_{\Delta}$ [*blaR1*(F284A)-*blaI*]	RN4220 with *pTX*$_{\Delta}$ that contains a functional *blaR1* with a F284A mutation and a WT *blaI*.	This Study
SFermS *pTX*$_{\Delta}$ 16	SFermS with the *pTX*$_{\Delta}$16 empty vector.	This Study
SFermS *pTX*$_{\Delta}$ [*blaR1*-*blaI*]	SFermS with *pTX*$_{\Delta}$ containing a functional *blaR1* and *blaI*.	This Study
SFermS *pTX*$_{\Delta}$ [*blaR1*(null)-*blaI*]	SFermS with *pTX*$_{\Delta}$ containing a functional *blaI* and a *blaR1* null mutant.	This Study
SFermS *pTX*$_{\Delta}$ [*blaR1*(null)-*blaI*(null)]	SFermS with *pTX*$_{\Delta}$ that contains null mutants of *blaR1* and *blaI*.	This Study
SFermS *pTX*$_{\Delta}$ [*blaR1*(F284A)-*blaI*]	SFermS with *pTX*$_{\Delta}$ that contains a functional *blaR1* with a F284A mutation and a WT *blaI*.	This Study

Supplementary Table 4: List of plasmids used for *S. aureus* growth assay.

Plasmid	Description	Reference
$pTX_{\Delta}16$	Empty vector	[3]
pTX_{Δ} [*blaR1*-*blaI*]	pTX_{Δ} containing a functional *blaR1* and *blaI*	This study
pTX_{Δ} [*blaR1*(null)-*blaI*]	pTX_{Δ} containing a functional *blaI* and a *blaR1* null mutant.	This study
pTX_{Δ} [*blaR1*(null)-*blaI*(null)]	RN4220 with pTX_{Δ} that contains null mutants of *blaR1* and *blaI*	This study
pTX_{Δ} [*blaR1*(F284A)-*blaI*]	pTX_{Δ} that contains a functional *blaR1* with a F284A mutation and a WT *blaI*.	This study

Supplementary Table 5: List of primers used for *S. aureus* growth assay.

Primer	Sequence	Purpose
blaR1-BamH1-for	ttaGGatCCatatttgaaagaaggtgtcaaaatggc	Cloning of *blaR1*-*blaI* into pTX_{Δ}
pTX_{Δ} -*blaR1*-I-Mlu1-rev	Cloning of *blaR1*-*blaI* into ptxD	Cloning of *blaR1*-*blaI* into pTX_{Δ}
pTX_{Δ} -*blaR1*-null-F	ttaGGatCCatatttgaaagaaggtgtcaaaacggctaaattac	Cloning of *blaR1*-*blaI* into ptxD, introduction of a M1T null mutation in *blaR1*
blaI-null-rev	tttcaacttgcttattggtcgtttaaaacacccattcc	Introduction of a M1T null mutation in *blaI*
blaI-null-for	ttttaaacgaccaataagcaagttgaaatatctatgg	Introduction of a null mutation in *blaI*
*blaR1*_F284A-Rev	tttttaccattTGCtgaatggcttaatatattttgttgg	Introduction of F284A mutation in *blaR1*
*blaR1*_F284A-For	atatattaagccattcaGCAaatggtaaaaaatcattactc	Introduction of F284A mutation in *blaR1*
